# Modeling Effects of RNA on Capsid Assembly Pathways via Coarse-Grained Stochastic Simulation

**DOI:** 10.1371/journal.pone.0156547

**Published:** 2016-05-31

**Authors:** Gregory R. Smith, Lu Xie, Russell Schwartz

**Affiliations:** 1 Department of Biological Sciences, Carnegie Mellon University, Pittsburgh, Pennsylvania, United States of America; 2 Joint Carnegie Mellon/University of Pittsburgh Ph.D. Program in Computational Biology, Pittsburgh, Pennsylvania, United States of America; 3 Computational Biology Department, Carnegie Mellon University, Pittsburgh, Pennsylvania, United States of America; University of Alabama at Birmingham, UNITED STATES

## Abstract

The environment of a living cell is vastly different from that of an in vitro reaction system, an issue that presents great challenges to the use of in vitro models, or computer simulations based on them, for understanding biochemistry in vivo. Virus capsids make an excellent model system for such questions because they typically have few distinct components, making them amenable to in vitro and modeling studies, yet their assembly can involve complex networks of possible reactions that cannot be resolved in detail by any current experimental technology. We previously fit kinetic simulation parameters to bulk in vitro assembly data to yield a close match between simulated and real data, and then used the simulations to study features of assembly that cannot be monitored experimentally. The present work seeks to project how assembly in these simulations fit to in vitro data would be altered by computationally adding features of the cellular environment to the system, specifically the presence of nucleic acid about which many capsids assemble. The major challenge of such work is computational: simulating fine-scale assembly pathways on the scale and in the parameter domains of real viruses is far too computationally costly to allow for explicit models of nucleic acid interaction. We bypass that limitation by applying analytical models of nucleic acid effects to adjust kinetic rate parameters learned from in vitro data to see how these adjustments, singly or in combination, might affect fine-scale assembly progress. The resulting simulations exhibit surprising behavioral complexity, with distinct effects often acting synergistically to drive efficient assembly and alter pathways relative to the in vitro model. The work demonstrates how computer simulations can help us understand how assembly might differ between the in vitro and in vivo environments and what features of the cellular environment account for these differences.

## Introduction

Understanding the detailed pathways by which virus capsids assemble during cellular infection is a challenging question that has been the focus of decades of study by experimentalists and theorists alike. The complexity and diversity of reaction pathways for each virus studied, a large variety of potential intermediates and the inability of any current experimental technologies to monitor assembly directly all present major obstacles to answering this question. Simulation methods have proven effective at offering a window into details of assembly that cannot be observed experimentally [1–21]. A variety of simulation techniques have been applied for this purpose, including ordinary differential equation (ODE) models [1–6], coarse-grained Brownian dynamics (BD) models [7–15], and discrete stochastic simulation algorithm (SSA) models [16–21]. We use the latter approach in the present work. While each method has distinct strengths and weaknesses, the SSA approach has filled an important niche in that it allows for fine-scale models of assembly at the level of single-particle interactions needed to study detailed assembly pathways (as in BD but not ODE models), computational feasibility of sampling large numbers of trajectories needed for computing pathway statistics and performing model-fitting (as in ODE but not BD models), efficiency over broad parameter ranges spanning typical in vitro and in vivo domains (as in ODE but not BD models), and parameterization in terms of small sets of easily interpretable interaction rate constants (as in ODE but not BD models). These advantages are crucial to the data fitting methods central to the present work as well as to gathering sufficient numbers of trajectories to characterize the consistent trends across highly stochastic pathway sets.

Traditionally, simulation methods have been useful for exploring possible trajectories of abstract models of assembly but have provided little guidance about detailed assembly pathways of any particular virus capsids due to the difficulty of learning quantitative model parameters that describe any particular virus' behavior. We previously developed an approach to combine discrete stochastic simulation of coarse-grained rule-based capsid assembly models with numerical optimization algorithms to learn kinetic rate parameters that best fit assembly simulation output to experimental data from real viruses [22,23]. This combination gave us the ability to learn detailed kinetic models and assembly pathways tuned to describe assembly of specific virus capsids, as measured by bulk in vitro light scattering data. Applying our method to three model icosahedral viruses—cowpea chlorotic mottle virus (CCMV), hepatitis B virus (HBV) and human papillomavirus (HPV)–yielded detailed simulations of subunit-level capsid assembly in vitro, revealing dynamics of assembly far more complex than had been captured by any prior theoretical or modeling studies.

While that work represented an important advance in learning assembly models fit to real viruses, it was nonetheless limited by the fact that models were by necessity learned from data on bulk assembly of purified coat protein in vitro, not assembly in the real living system. Our ultimate goal is to characterize assembly in vivo, but there are as yet no experimental technologies to gather quantitative time-series measurements of assembly progress in vivo to which we might fit simulation models. We have therefore pursued an alternative approach of attempting to "correct" in vitro models to account for known differences between the cellular and test-tube environments likely to influence assembly kinetics or pathways. We previously applied this approach to project likely effects of cellular levels of macromolecular crowding on capsid models learned in vitro [24], revealing how crowding could act to accelerate and stabilize assembly in vivo while avoiding kinetic traps predicted by earlier theoretical models.

This study follows a comparable approach to that earlier work to examine another important difference between in vitro and in vivo assembly environments for many viruses—the presence of nucleic acid—with specific focus on the process of concurrent assembly and packaging typical of RNA viruses [25–28]. We specifically examine CCMV, a single stranded RNA (ssRNA) icosahedral T = 3 virus that has been an important model system for capsid assembly studies [29]. To deal with the computational challenges of modeling RNA effects on the timescale of capsid assembly, we use a combination of simulation and analytical modeling to attempt to project different likely effects of RNA on CCMV assembly. As with our prior work on projecting crowding effects on assembly [24], we use a model of purified coat protein assembly learned from in vitro light scattering data and then build in fast corrections to that model to account for likely influences lacking from the experimental data. This approach provides a way to take advantage of the high efficiency of the stochastic simulation methods, and the data-fitting technology they enable, to gather statistics on detailed pathway usage of a data-fit model of a real virus.

Experimental results on RNA packaging [30–36] have opened a door to understanding the physical effects of RNA on capsid assembly, by, for example, providing information on optimal genome lengths for capsid stability and packaging efficiency [33,34], structural data on the localization of RNA within the capsid [36], or tests of presence of certain intermediates during assembly [35]. There has been ongoing debate within the field about the relative importance of sequence specificity in RNA packaging, with evidence from some model systems supporting the importance of specific packaging signals on the RNA that act to recruit capsid proteins and potentially initiate capsid assembly [30–32]. In HIV, for example, it was found that changes in RNA packaging signal binding specificity can act as a regulatory element in viral synthesis [37]. However, other viruses show much greater flexibility in what can be encapsidated provided certain charge and size requirements are met [33,34]. Various prior theoretical and computational approaches have been applied to understand the effect of RNA on capsid assembly, including analytical models [38–46] and inclusion of RNA strands in coarse-grained assembly simulations [20,47–52]. Coarse-grained Brownian models have proven effective at showing diverse assembly pathways and finding biologically-relevant associations between unique pathways and corresponding assembly conditions, such as varying disorder in assembly intermediates being dependent upon salt concentration [48]. Furthermore, they have provided fascinating insights into the interplay of RNA and larger assembly intermediates and even how assembly reaction kinetics might be altered under the presence of RNA. There are limitations, however, to the detail one can capture when only considering small numbers of larger intermediates. Our previous studies have suggested that key features of assembly, such as nucleation, are driven by complex cascades of small-scale events below the resolution of what past coarse-grained explicit simulations including nucleic acid have been able to examine [23,24]. In the case of CCMV, we have found the majority of assembly reactions consist of nucleation via cascades of interactions of small oligomers followed by elongation via addition of single dimers and trimers-of-dimers. The coarser structural models required to make Brownian simulations tractable would make it impractical to simulate these pathways. Furthermore, the stochastic simulation approach employed in the present paper, by avoiding explicit modeling of diffusion, is much more scalable to changes in concentration, allowing them to run efficiently across trajectories with orders of magnitude difference in assembly rates. The tradeoffs between the more simplified models of physics but more detailed models of assembly pathways allowed by our methods, versus the more detailed physics models but simplified pathway sets of explicit particle models, create a niche for each model in providing a window into the real phenomenon. The level of assembly detail provided by our method, in particular, allows unprecedented insight into how the presence of RNA not only may affect the kinetic rates of assembling capsids but also how these changes can alter the fine-scale pathways by which a CCMV capsid assembles.

CCMV has four RNA strands that can potentially be encapsidated: RNA1 and RNA2, which are each encapsidated individually, and RNAs 3 and 4, which are encapsidated together. We specifically use RNA1 for our model but make no explicit assumptions about specific packaging signals. To model electrostatic effects of RNA, we apply a Flory theory [53] to calculate free energy changes induced by encapsidating a charged RNA polymer. We separately examine four individual likely influences of RNA on assembly: the energy of RNA-RNA interactions during packaging, the entropic cost of confining the RNA polymer within the CCMV capsid, the energy of charged interaction between the capsid proteins and the RNA polymer, and the increased local concentration enabled by packing coat monomers on a single RNA strand. We apply analytical models of these effects to adjust kinetic rate parameters experimentally fit to bulk CCMV assembly data and examine how these different RNA effects, individually and in concert, might modify CCMV capsid assembly. The results reveal a complex interplay of influences that collectively greatly enhance assembly while altering both kinetics and pathway usage relative to the in vitro model.

## Materials and Methods

### Capsid simulation method

We have previously developed a rules-based discrete event stochastic simulator called the Discrete Event Simulator of Self-Assembly (DESSA) [54] to model the process of capsid assembly from individual subunit building blocks through individual association and dissociation events into completed capsids. Simulated assembly is governed by simple biochemical rule sets specifying the geometries of the subunits, three-dimensional positioning of binding sites and the specificities and on- and off-rates of binding events between binding sites. With respect to CCMV, all subunits that compose the final capsid structure for the virus are chemically identical. Different conformations of these subunits, however, bind together to form the completed capsid structure. The combination of conformations, their shapes, and their binding site specificities imply an overall geometry to the completed capsid. DESSA samples among all possible bond formation (association) and breaking (dissociation) events at each step in the simulation using a variant of the stochastic simulation algorithm (SSA) [55,56]. For each potential reaction event, a corresponding event time is calculated by sampling an exponential distribution whose mean value is determined by the given kinetic rate for that interaction. The minimum-time event among all possible next reactions is selected based on these sampled times, yielding a kinetically correct sample of possible trajectories from among the full ensemble of possible trajectories implied by the rule set. Once an event is selected, the simulator evaluates the reaction to ensure it is not sterically hindered and, if not, implements it and updates the event queue with times for any possible reactions enabled by the new event. A simulation ends when either all potential events have been exhausted or a predetermined time limit has been reached. We model CCMV assembly via dimers of coat proteins as the individual subunits since the experimental data also involved in vitro assembly from coat dimers [29]. The DESSA simulator and sample input xml files are publicly available at https://github.com/rsschwartz/Dessa2.0.

We assumed kinetic rates in the hollow capsid case to be those derived from a previous study [23] in which we used numerical optimization to learn parameters that fit in vitro experimental static light scattering data [29] to light scattering curves generated based upon our DESSA simulation output [23]. The data-fitting algorithm is based upon a local optimizer of parameter sets to a quality-of-fit RMSD measure that uses an adaptive interpolation between gradient-based and quadratic response surfaces to iteratively converge on the location of a local minimum. This local optimization is in turn used as the basis of a heuristic global search. The global search proceeds by an initial scan of a reduced parameter space derived by assuming all on-rates in the system are equal and all off-rates are equal. We then perform a series of local searches, each using the local optimum of the previous search as an initial guess for a search in an expanded parameter space in which two previously equal off-rates are allowed to vary independently. This process repeats until each off-rate is independently fit.

The following sections describe modifications to the best-fit kinetic rate parameters for the hollow capsid case described above. These modifications reflect changes in free energy or capsomer concentration dependent upon different physical effects of the presence of RNA within an assembling capsid. We specifically model the case of a single CCMV capsid encapsidating RNA 1, which consists of 3171 nucleotides.

### Modeling Nucleic Acid Effects

To better understand the specific ways in which RNA might influence CCMV capsid assembly, we subdivided estimation of RNA effects on model rate constants into four contributions: 1) RNA-RNA interactions (subsequently abbreviated RNA-RNA), 2) Entropy of RNA chain compression (subsequently abbreviated Compression), 3) RNA-protein interactions (subsequently abbreviated RNA-protein), and 4) local concentration of coat on the RNA (subsequently abbreviated Concentration). We then apply analytical models to estimate the strengths of each contribution, an approach adapted from related work of Zandi and van der Schoot [39]. While analytical models of each effect involve various approximations and assumptions, they provide sufficient guidance to give a theoretical projection of how these forces might act individually and in concert to alter overall kinetics and pathways of assembly. They allow us to ask some fundamental questions about the possible differences between capsid assembly in vivo versus in vitro: specifically, is it plausible that energetic influences of RNA, modeled as a generic polyelectrolyte, are sufficient to produce a qualitative change in assembly pathways for an RNA virus, and, if so, by what mechanisms could they produce such a change? While the answers to those questions will necessarily fall short of making a definite pronouncement about what does happen in the real system, they can provide guidance to future theoretical and experimental studies to answer these questions more definitively. RNA-protein and Concentration both cause an increase in equilibrium constant for CCMV, whereas Compression and RNA-RNA cause a decrease in equilibrium constant. We apply RNA-RNA and RNA-protein effects to both the on and off rates evenly, while the effect of Compression and Concentration are applied solely to the on rates. These corrections are then applied to our previous best fit rate parameter values to represent the individual nucleic acid effects. We consider each contribution in turn:

#### RNA-RNA interactions (RNA-RNA)

We treat the RNA within the capsid as a polymer of individual Kuhn segments of RNA, each of Kuhn length *b*. Each Kuhn segment represents the length of nucleotides necessary to model the polymer as freely jointed in solution. For semi-flexible or worm-like chains (WLC), the Kuhn length is approximately equal to double the persistence length of the polymer [57]. The persistence length of RNA in a 1M monovalent salt solution has been estimated to be 1.3 nm [58], and thus *b* = 2.6 nm. The RNA is not solely confined to the entire interior of the capsid, however. The RNA is modeled as a thin layer inside the capsid wall in the case of CCMV RNA 1, as suggested by prior cryo-EM study of RNA-containing CCMV [59]. We estimate a thickness *D* ≈ 1.18 *nm* by extrapolation from similar studies on other virus systems [60]. We then calculate the Flory free energy for the polymer chain via the formula [61]

$$F_{RNA}\approx k_{B}T\left(\frac{l^{2}}{Nb^{2}}+\frac{1}{2}v\frac{N^{2}}{V}\right)$$ (1)

accounting for entropic and excluded volume interaction effects respectively. The excluded volume interaction term is reduced to reflect the presence of counterions on the RNA strand. *N* is the number of Kuhn segments, *V* is the pervaded volume of the RNA calculated as the volume within the capsid filled by the RNA:

$$V=\frac{4}{3}\pi (R^{3}-{\left(R-D\right)}^{3})$$ (2)

Here, *R* = 10.5 nm is the inner radius of the capsid. *l* is defined to be the end-to-end distance of the RNA polymer which we calculate to be the maximum distance traversed within the volume of the RNA inside the capsid, i.e., *l* = π*R* ≈ 32.99 nm. We define *v* as the excluded volume of a given Kuhn segment of the RNA. We calculate the excluded volume, following [39], via the formula:

$$v\approx \frac{\pi b^{3}}{48}+\frac{64\pi \lambda_{B}^{3}\lambda_{D}^{2}}{b^{2}}\approx 3.537nm^{3}$$ (3)

where *λ*_*B*_ ≈ .7*nm* is the Bjerrum length and *λ*_*D*_ ≈ .3*nm* is the Debye screening length in 1M monovalent salt [39]. The ratio of excluded to pervaded volume captures the amount of flexibility available to chain bending and thus influences the entropy of chain folding. Thus, for CCMV capsids assembling around RNA 1, which is a single strand of 3171 nucleotides, *N* ≈ 414.669 and *F*_*RNA*_ ≈ *k*_*B*_*T*(.388+208.57) = *k*_*B*_*T*(208.958). This energy yields a multiplicative equilibrium constant *K*_*EQ*_ of $e^{\frac{-208.958}{180}}=.3132$ [61]. We would expect this energy to correspond to yield effects on both on- and off-rates due to both the time required for the RNA chain to find an appropriate fold consistent with the final shell structure and to the destabilizing effects holding tightly packed RNA in this configuration. In the absence of specific evidence about how the energetic effects would be distributed to on- versus off-rates, we approximate these kinetic effects by attributing the equilibrium change equally to on and off rates, multiplying the rate of binding events by $\sqrt{.3132}=.5597$ and dividing the rate of dissociation events by .5597 as well. The model further assumes that these contributions are distributed equally to each coat monomer adding to the shell, effectively assuming the RNA adopts its final configuration in the vicinity of the assembling capsid as the capsid forms.

#### Entropy of RNA chain compression (Compression)

We calculate the free energy change from RNA compression as a separate entropic cost from that described above [61]. The RNA is not solely confined to the entire interior of the capsid [36], and so we must constrict the volume confinement further to reflect the nature of the thin layer of RNA within the capsid surface. Thus, the free energy of confinement is calculated by

$$F_{conf}\approx k_{B}T\frac{Nb^{2}}{R^{2}-{\left(R-D\right)}^{2}}\approx k_{B}T(119.86)$$ (4)

Here, R is the inner radius of the capsid and *Nb*^2^ / (*R*^2^ − (*R* − *D*)^2^) represents the number of compression blobs [58] for the RNA polymer confined inside the capsid. A compression blob represents a random walk of Kuhn segments that fill the cavity of compressed space. That is to say, a length scale where the polymer chain can be expected to be unperturbed by an external force of interest; in this case, compression of the polymer chain. *R*^2^ / *b*^2^ represents the number of Kuhn segments comprising a single compression blob for a spherical cavity of radius *R*. Because we further confine the RNA to a thickness of width *D* within the spherical capsid, the number of Kuhn segments comprising a single compression blob is reduced to *R*^2^ / *b*^2^ − (*R* − *D*)^2^ / *b*^2^ = (*R*^2^ − (*R* − *D*)^2^) / *b*^2^. The resulting term effectively captures the entropy of possible ways of distributing the volume of the RNA chain within the larger volume of the capsid shell. Following the same method as above [62], we convert *F*_*conf*_ into a *K*_*EQ*_ scaling factor of $e^{\frac{-119.86}{180}}=.5138$ which we attribute solely to on rates. The attribution of this contribution to a reduction in on-rate would be consistent with a model in which time is required for RNA folding to find a configuration that allows capsid assembly to proceed.

#### Free energy of RNA-protein interactions (RNA-Protein)

To understand the free energy associated with RNA-protein interaction, we have to take into consideration the attractive force between the negatively-charged RNA polymer and the positively charged capsid proteins. Here, instead of treating Kuhn segments as our basic monomers, we will treat a single nucleotide as a monomer as that reflects a single charge interaction with the capsid proteins. We will determine the energetics of this interaction via the Flory theory of an adsorbed chain to a charged surface assuming that monomers (nucleotides) are uniformly distributed within the thickness of the RNA inside the capsid [62]. Thus, we calculate the free energy associated with RNA-Protein charge interaction following [61]:

$$F_{ch}\approx -k_{B}T\delta M\frac{r}{D}$$ (5)

Here, *δ* represents the adsorption energy per nucleotide-capsid protein contact, which we set at 0.5 to reflect the presence of counterions on the RNA polymer, M = 3171 is the number of nucleotides in the RNA polymer, and r = .34 nm is the length of a single RNA nucleotide. Thus, $F_{ch}\approx -k_{B}T\frac{1}{2}3171\left(\frac{.34}{1.18}\right)=k_{B}T(-456.84)$. We convert this into a *K*_*EQ*_ scaling factor of $e^{\frac{456.84}{180}}=12.6543$. We would anticipate that this effect would have components acting both to accelerate the on-rate and reduce the off-rates both by charge-charge interactions helping draw together coat monomers via RNA to the assembling shell and by the stabilizing effects of these same charge-charge interactions. We approximate these combined effects by dividing the free energy contribution evenly between the on and off rates, multiplying the rate for bond forming events by 3.5573 and dividing the rate for bond breaking events by 3.5573 as well.

#### Local coat protein concentration by RNA (Concentration)

The in vitro capsid assembly experiments we have previously used to learn simulation parameters have capsid protein concentrations between 5 and 20 μM [23,59]. We would expect the effective concentration to be vastly higher for coat proteins aggregated onto an initially disordered RNA strand. We estimate this local concentration by treating the RNA as a polymer in a weak confinement regime (WCR) [62]. We use the formula $R_{3}(radius)=aN^{v_{3}}$ where a is the length of an individual nucleotide, 3.4Å, N is the number of nucleotides in the polymer and *v*_3_ is the Flory exponent, which is set to 0.6 to approximate the expected value for a good solvent.

In the case of CCMV, *R*_3_ = 428.7426*Å* and then the corresponding volume is *V* = 3.013 × 10^8^ *Å*^3^. Previously, we used in simulations an experimental concentration of 15.6 μM of CCMV capsid proteins [23,24]. This reflects 9.39432 × 10^21^ molecules/m3. For a volume of 3.013 × 10^8^ *Å*^3^, we would have 3.1013 dimers at 15.6 μM. In order to have 90 dimers present in that volume, we would need an increase in concentration to $\frac{90}{3.1013}\times 15.6\mu M=452.71\mu M$. To reflect this increase we multiply the on rates by $\frac{90}{3.1013}=29.02$ in our simulation input, a change that follows from standard mass-action kinetics models to reflect the greater rate of pairwise collisions in higher concentrations.

### Simulation Experiments and Analysis

**Fig 1:**
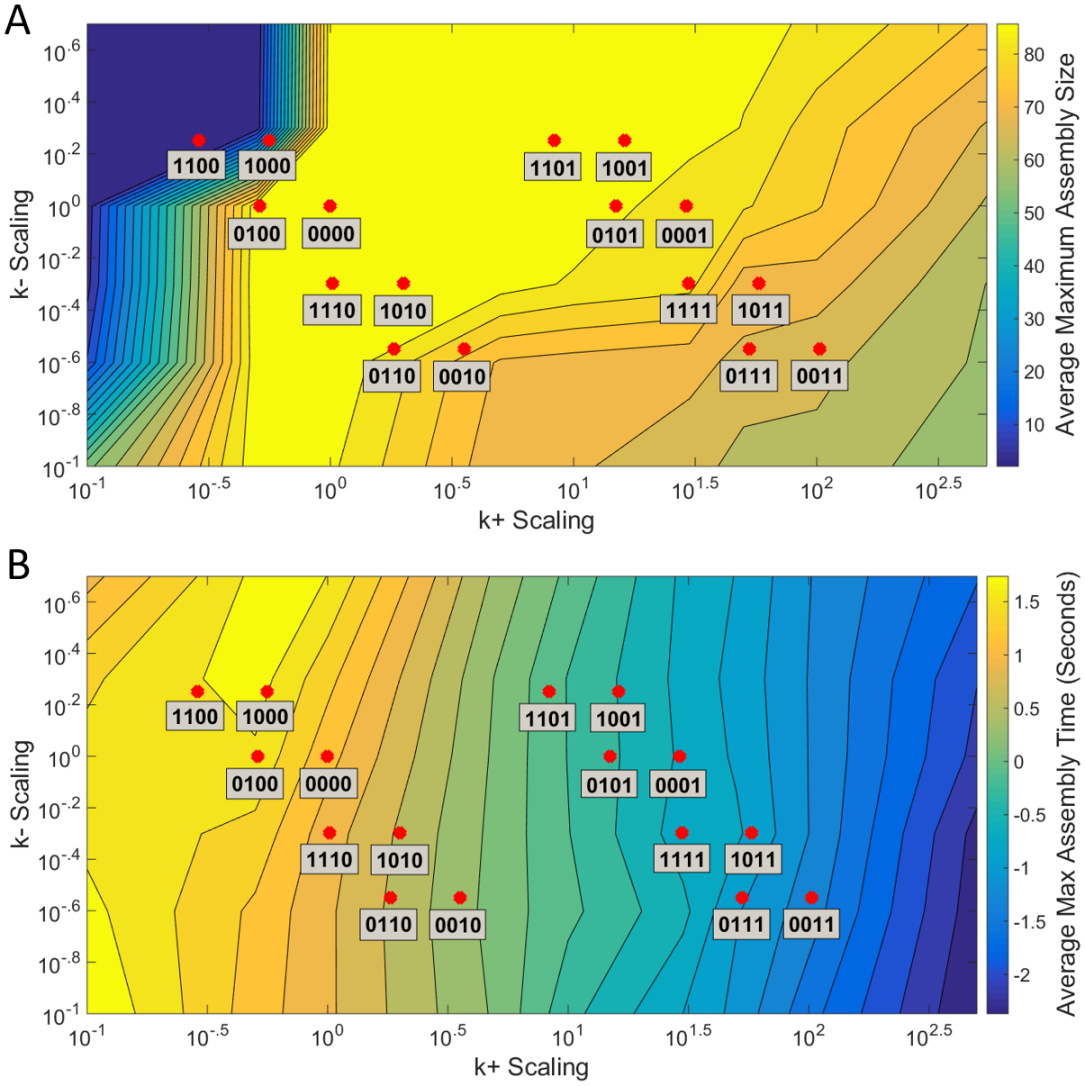
Contour plots of maximum assembly size and assembly time over the space of rate parameters. (A) Contour plot of the average maximum assembly size for 54 rate parameter grid points and 16 RNA effect combinations (red dots). The colorbar shows average maximum assembly size. A completed CCMV capsid consists of 90 subunits. (B) Contour plot of the average time to reach a maximum assembly size for a simulation over the same grid points and effect combinations. The colorbar shows time in seconds. The four-digit binary codes for simulations are as explained in Table 1, with a zero in each bit corresponding to absence of a given RNA effect and a one in that bit to presence of the corresponding effect. The first digit represents RNA-RNA, the second Compression, the third RNA-protein, and the fourth Concentration.

We produced corrected parameter files, dividing effects into the four categories discussed above: RNA-RNA, Compression, RNA-protein and Concentration. We further examined all sixteen possible combinations of these individual factors. We summarize the possible combinations of effects and the resulting corrections to the reaction rate parameters of the simulations in Table 1, using a four-digit binary code to represent the combination of effects in any given parameter domain. The first digit represents RNA-RNA, the second digit represents Compression, the third digit represents RNA-protein and the fourth digit represents Concentration. A value of 1 means that effect is turned on and a value of 0 means that effect has been turned off. These modifications to the rate constants described in Table 1 are also depicted visually in Fig 1, superimposed on contour plots of mean assembly sizes and times as a function of the rate modifications. Rate constant modifications for both parameters are spread over multiple orders of magnitude.

**Table 1:** Corrections to equilibrium constants, on-rates, and off-rates for all possible combinations of four RNA effects.

CCMV1	*K*_*eq*_(*mol*^−1^*m*^2^)	k_+_ (mol^-1^m^2^s^-1^)	k_-_ (s^-1^)
0000 –Hollow	1	1	1
1000 –RNA-RNA	.3132	.5597	1.787
0100 –RNA Compression	.5138	.5138	1
0010 –RNA-Protein	12.65	3.557	.2812
0001 –Concentration	29.02	29.02	1
1100	.1609	.2876	1.787
1010	3.962	1.991	.5025
1001	9.089	16.24	1.787
0110	6.500	1.828	.2812
0101	14.91	14.91	1
0011	367.1	103.2	.2812
1110	2.036	1.023	.5025
1101	4.670	8.345	1.787
1011	115.0	57.77	.5025
0111	188.6	53.04	.2812
1111	59.08	29.68	.5025

For brevity, we use a four digit binary code to reflect the combinations of effects. 0000 represents a hollow capsid with no RNA effects modeled, while 1111 represents all four effects included. The first digit represents the free energy of RNA-RNA interactions (RNA-RNA), the second digit represents compression of the RNA strand inside the capsid (Compression), the third digit represents RNA-capsid protein interactions (RNA-protein), and the fourth digit represents local concentration of capsid proteins on the RNA polymer (Concentration).

We ran 100 simulation trajectories for CCMV for each of the 16 combinations of effects. In our previous work, we included enough capsid proteins to produce multiple completed capsids [23,24]; however, in this case, since we are looking at the specific process of one capsid assembling about its RNA, we limited the number of initial subunits to an amount just sufficient to assemble a single capsid, 90 subunits in the case of CCMV. Each simulation ends when either a completed capsid consisting of all initial subunits is formed, or a time limit of 100 seconds has been reached. This predetermined time limit was empirically determined to allow simulations to reach a state of pseudoequilibrium.

We apply a variety of analysis and visualization methods to study simulation trajectories individually and in aggregate. First, we generate simulated light scattering curves, comparable to the in vitro experimental data from which models were learned, to provide an overall visualization of assembly progress over time for each condition described above. We then derive summaries of assembly pathway usage in the form of matrix plots of frequencies of possible binding interactions averaged across all trajectories and time-series of mass fractions of different sizes of species versus time for single trajectories. To construct these frequency matrix plots, we count all association events that occur across all repetitions of each simulation input parameter set and produce a matrix with one row or column for each subunit size contained in a completed capsid. We place in position (*i*,*j*) of the matrix the count of all binding events involving an assembly of *j* subunits producing an assembly of *i* subunits normalized by the total number of association events involving the production of an assembly of *i* subunits. Thus, each position in the matrix contains a frequency between 0 and 1. For example, if 90% of all 10mers are formed by the binding of a 9mer and a monomer, then the values for the 1^st^ and 9^th^ columns in the 10^th^ row are .9. To construct mass fraction plots, we record after each simulation event the quantity of each assembly size (from monomer to complete capsid) present in the simulation at each time point. We then scale each of these assembly size counts by the total number of subunits present in the assembly (e.g., we multiply the count of pentamers by 5 to reflect the five monomers present in each pentamer, yielding a count of the total number of subunits present in pentamers). We finally normalize each value such that it represents the frequency, between 0 and 1, instead of the total count of subunits in each assembly size. If there are 450 initial subunits in the simulation, each total scaled count value is divided by 450 to reflect the fraction of subunits found in each species size. We plotted these mass fraction values versus simulation time for each potential assembly size. We further generated movie files, provided in the Supporting Information as S1–S4 Movies, visualizing the complete sequence of binding and dissociation events involved in a single trajectory for each parameter setting.

## Results and Discussion

Table 1 shows the corrections to the equilibrium constant as well as on and off rates under the individual changes based upon the four effects modeled: RNA-RNA, Compression, RNA-protein, and Concentration, as well as different potential combinations of these effects. We would expect Concentration and RNA-protein to yield net positive (assembly-promoting) contributions and RNA-RNA and Compression to yield net negative (assembly-inhibiting) contributions. We examine all sixteen possible combinations of presence or absence of the four effects running 100 assembly simulations for each combination. To understand how these sixteen effect combinations relate to the overall space of on and off rate scaling parameters, we conducted a grid search of scaling parameter combinations covering on-rate scaling values between .1 and 500 and off-rate scaling values between .1 and 5, both ranges large enough to include all combinations detailed in Table 1. The grid examined on rate scaling values of .1, .5, 1, 5, 10, 30, 50, 100 and 500, and off rate scaling values of .1, .25, .5, 1, 2 and 5. At each of the 54 grid points, we conducted 100 simulations, applying on and off rate scaling factors to the best fit rate parameters for CCMV capsid assembly determined in our previous work [23] and equivalent to the ‘0000’ case in Table 1. For each simulation at the 54 grid points and 16 RNA effect combination points, we determined the maximum assembly size achieved and the time in silico required to reach that maximum assembly size. We then calculated averaged values for each of these measures over all simulations at each data point.

Fig 1 shows contour plots of these two values: average maximum assembly size (A) and average time to reach maximum assembly size (B). We highlight the 16 RNA effect combination locations on the contour plots (red dots). With respect to maximum assembly size, there’s a large central vein in the plot where simulations consistently build completed capsid structures. This includes the hollow capsid (0000) case as well as a number of other cases: 0100, 1110, 1010, 0110, 1101, 1001 and 0101. The combined RNA effects case (1111) still very frequently builds completed structures. The average maximum assembly is still over 80 (compared to a completed capsid consists of 90 subunits). The 0001 case similarly is still very effective at building capsids, with the 0010 and 1011 cases somewhat diminished in their assembly production. There are four extreme cases: 1100 and 1000 in the upper left corner are incapable of building more than small intermediates on average while 0111 and 0011 in the lower right corner can only, on average, produce 60mers. This suggests that CCMV capsid assembly is sensitive to perturbations on both extremes of the parameter space although fairly insensitive around most parameter combinations, including the no-effects and all-effects cases. This result suggests that minor changes to the energy equations or attributions of energy to on- versus off-rates are unlikely to greatly affect rate or fidelity of assembly. With respect to assembly time, the relationship is a little more straightforward. As on rates increase, the speed to reach the maximum assembly size reached in a simulation increases as well. In this case, the combined RNA effects case (1111), while still being able to very frequently produce completed structures, produces capsids at a rate on average 59 times faster than the hollow capsid case (0000).

**Fig 2:**
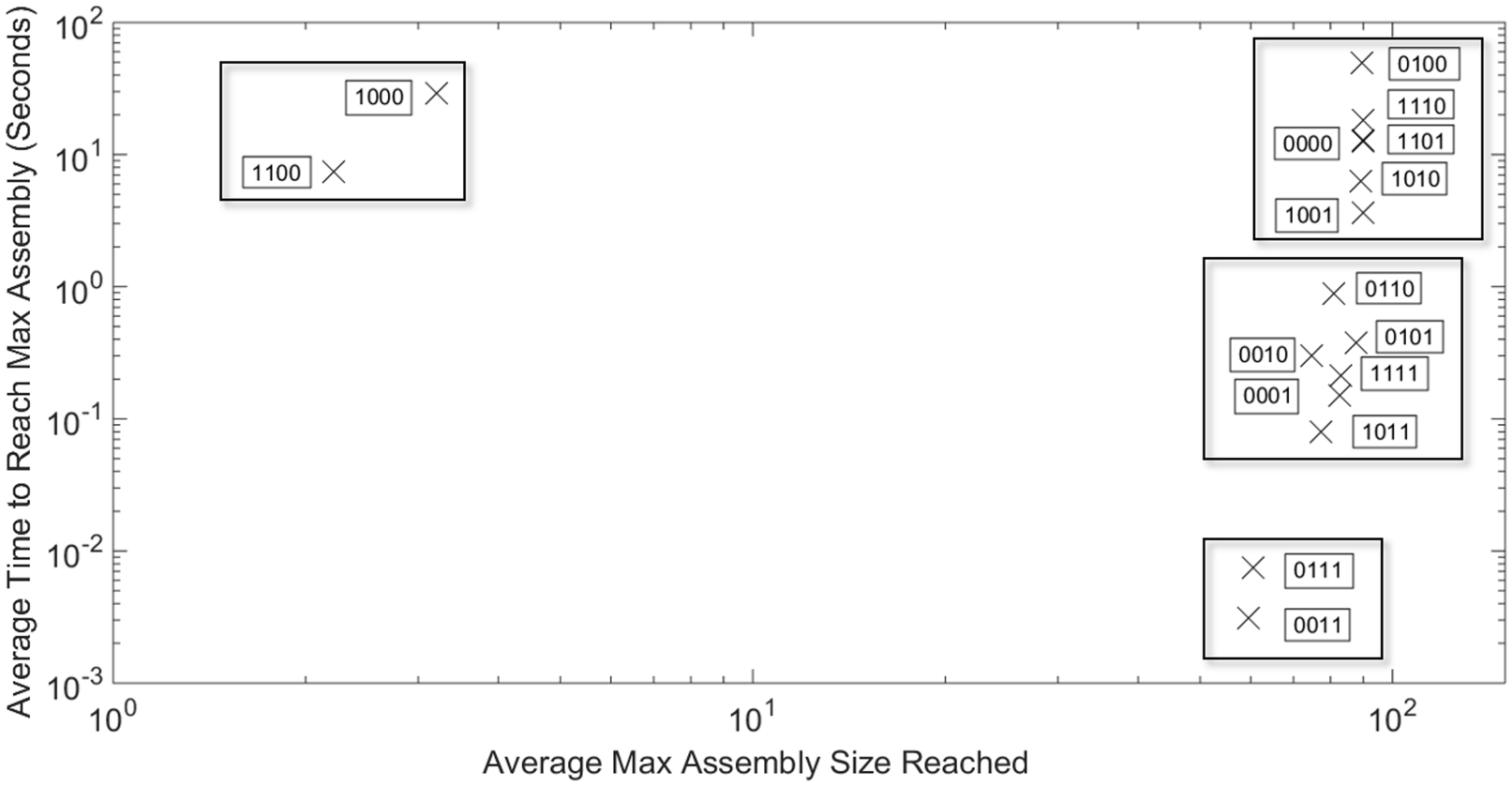
Comparing maximum assembly size and assembly time. Log-scale scatter plot of maximum assembly size versus the time to reach that assembly size averaged over 100 simulation runs for each RNA effect combination. Boxes surround clusters of RNA effect combinations that have similar relationships between maximum assembly size and assembly time. The four-digit binary codes for simulations are as explained in Table 1, with a zero in each bit corresponding to absence of a given RNA effect and a one in that bit to presence of the corresponding effect. The first digit represents RNA-RNA, the second Compression, the third RNA-protein, and the fourth Concentration.

To allow us to delve deeper into the relationship between maximum assembly size and assembly time for each RNA effect combination, Fig 2 provides a log-scale scatter plot of these two values for each effect combination. Error bars for each dimension appear as S1A and S1B Fig. What we find can be empirically clustered into four distinct qualitative classes of effect combinations with respect to how they modify the effectiveness of capsid assembly. These classes are enclosed in separate boxes in Fig 2. The box in the upper right corner reflect effect combinations that behave similarly to the hollow capsid (0000) case, where completed capsids (90 monomers) are formed consistently. The middle-right box below it shows effect combinations that cluster with the combined effects (1111) case where completed capsids are still formed with high fidelity, but at a much faster rate. The box containing combinations 0111 and 0011 in the lower right shows simulations in which assembly begins quickly, but gets kinetically trapped with final products much smaller than a completed capsid. Lastly, the box containing effects combinations 1100 and 1000 proceeds slowly and produces minimal assembly on the timescales examined. Box plots detailing the variability in each of these numbers over the hundred simulations are included in S1 Fig.

Based upon these classifications, the remainder of the results are divided into two sections. First, we focus on four representative combinations, one from each of the boxes in Fig 2: A) hollow capsid (no RNA effects, code 0000), B) the combination of the two negative effects (RNA-RNA and Compression, code 1100), C) the combination of the two positive effects (RNA-protein and Concentration, code 0011), and D) the combination of all four RNA effects (code 1111). The remaining twelve combinations each behaves similarly to one of these four representative combinations. Full results for these twelve cases are provided in the following results subsection.

### Analysis of Four Representative RNA Effect Combinations

#### RNA effects on bulk assembly kinetics

**Fig 3:**
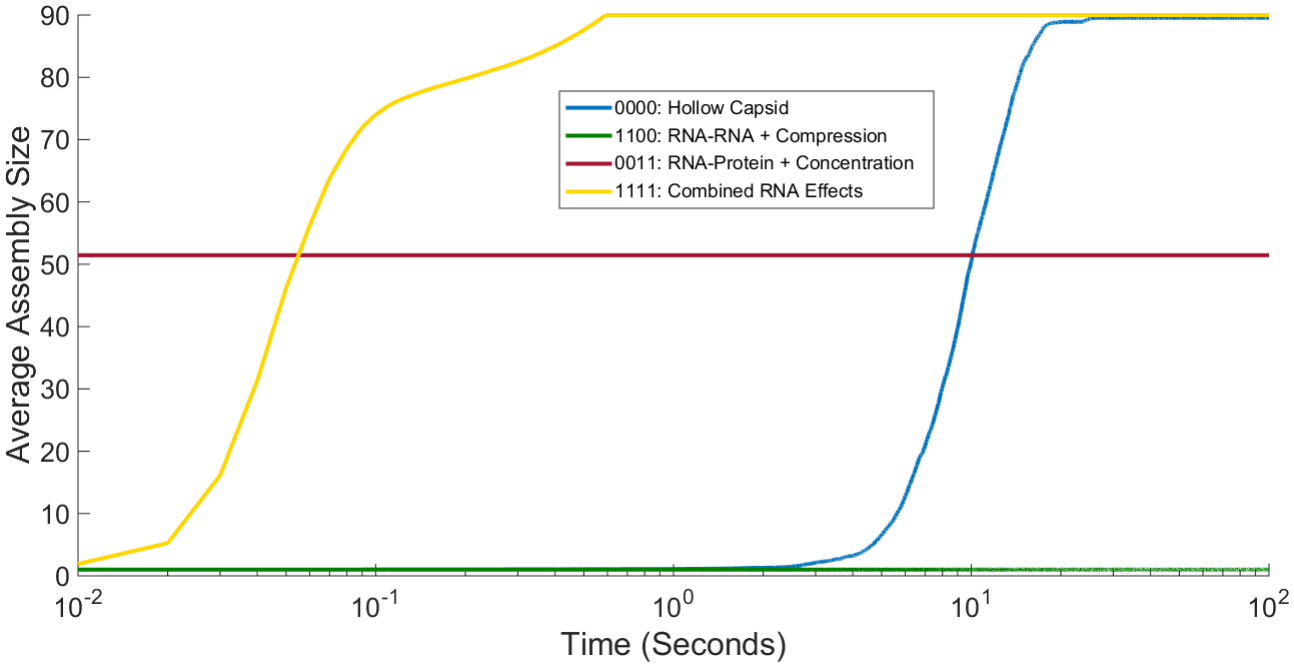
Simulated light scattering curves for CCMV capsid assembly under different representative combinations of RNA effects. Plot comparing simulated light scattering curves for CCMV capsid assembly averaged over 200 individual simulation trajectories for four representative combinations of RNA effects: hollow capsid (no effects considered), the two negative RNA effects (Compression + RNA-RNA), the two positive RNA effects (RNA-Protein + Concentration) and the combination of all four RNA effects. Time on the x axis is shown on a log scale.

Fig 3 shows simulated light scattering curves for CCMV under the four representative combinations of RNA effects. The hollow capsid curve behaves similarly to previous studies under no RNA effects [23,24], consistent with an initial lag phase followed by a sudden nucleation event then rapid growth to completion. The combined RNA effects curve also goes to completion but on a far faster time scale with no observable lag phase. In contrast, the other two cases in Fig 3 show unsuccessful assembly, but via two different failure modes. Combining the two negative effects, Compression and RNA-RNA, abolishes any significant assembly, resulting in a simulated light scattering curve that remains barely above the origin. Combining the two positive effects, RNA-protein and Concentration, produces rapid growth but plateaus at approximately half complete. This is a profile consistent with a regime observed when nucleation-limited growth breaks down, in which large intermediates assemble quickly and deplete the pool of free monomers, leading to a kinetically trapped system of partially assembled shells.

Additional combinations of parameters, examined in the subsection "Analysis of Twelve Remaining RNA Effect Combinations" below, seem to group approximately into one of the four paradigms observed here: A) slow, successful assembly; B) lack of assembly; C) fast, kinetically trapped assembly; or D) fast, successful assembly.

#### RNA effects on individual assembly trajectories

**Fig 4:**
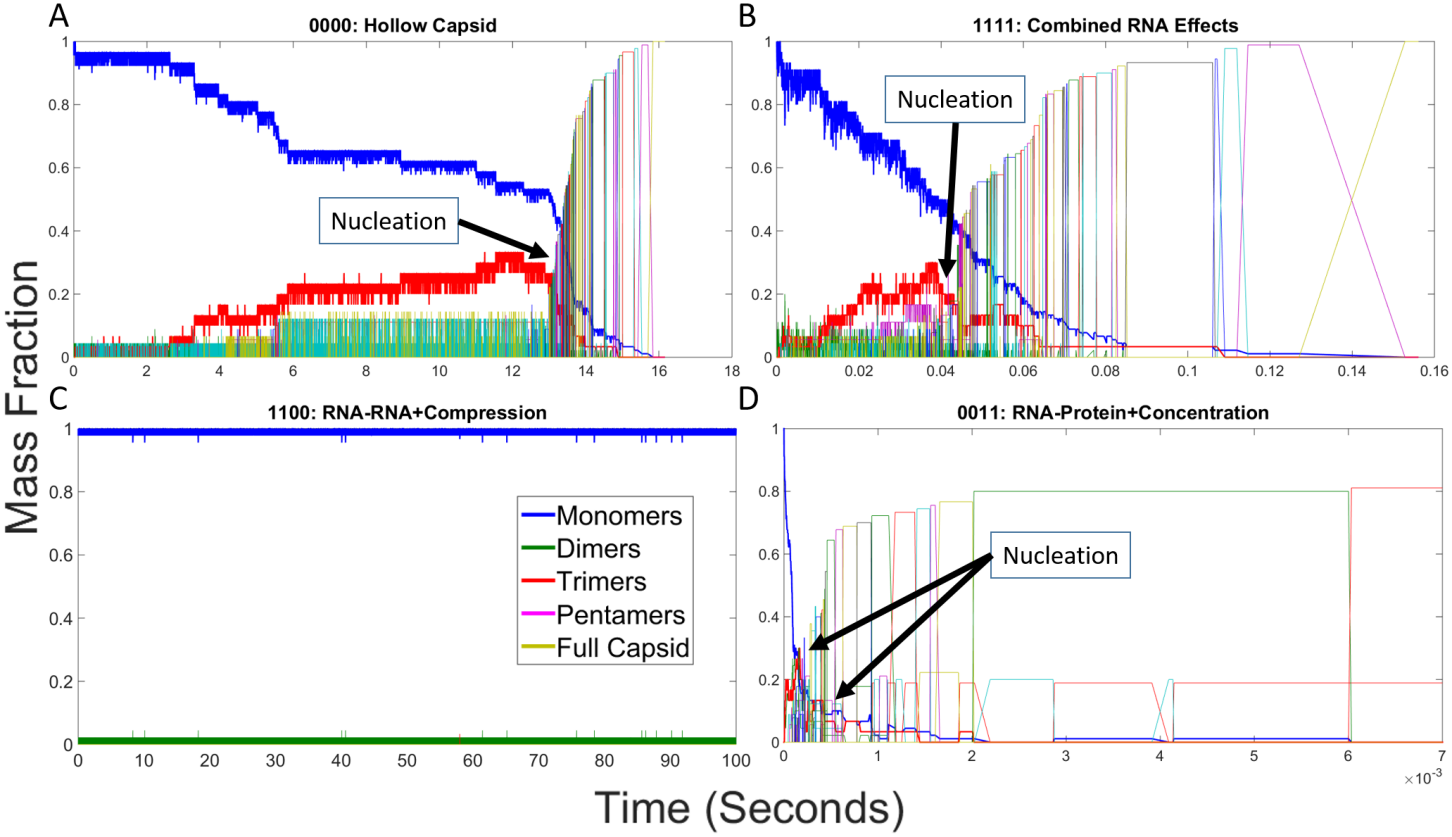
Mass fraction plots for representative combinations of RNA effects. Mass fraction plots for (A) hollow CCMV capsid assembly, (B) CCMV capsid assembly with all combined RNA effects, (C) CCMV capsid assembly under both negative effects (1100), and (D) CCMV capsid assembly under both positive effects (0011). Each plot measures the mass fraction of each potential assembly size from individual monomers to completed capsids at each time point in a single simulation run. Approximate locations of putative nucleation events are labeled in each plot. Note that the time axis is on a different scale for each plot due to the very different timescales of the assembly reaction under the different effects models.

We next examined individual trajectories for each parameter combination to better understand how low-level interactions give rise to altered assembly pathways and thus the high-level kinetic profiles observed in Fig 3. We constructed mass fraction plots from individual simulation runs for each combination of RNA effects. Each mass fraction plot shows the fraction of capsid proteins in assemblies of each potential size at any given point during the simulation trajectory. Fig 4 shows mass fraction plots for the four representative cases we highlight: hollow capsid (Fig 4A), combined RNA effects (Fig 4B), Compression + RNA-RNA (Fig 4C) and RNA-protein + Concentration (Fig 4D). Fig 4A initially shows a prolonged latency period where only small assemblies are produced, notably a pool of trimers (shown in red) building up in concert with a slow decline in the overall pool of monomers (shown in blue). This is followed by a sudden cascade of events then rapid completion of the capsid, consistent with a nucleation-limited growth mechanism observed for this model in our prior studies [23,24]. Fig 4B shows that the combination of all four events yields a qualitatively similar growth profile to the hollow capsid but on a timescale approximately 200-fold faster. One notable feature of this profile is that the lag period and elongation period are approximately equal in length, which would normally be expected to abolish nucleation-limited growth and move a system into a kinetically trapped regime. We further note that an additional pool of pentamers builds up in the lag phase and drains away in the elongation phase, alongside the trimer pool that was observed in the empty capsid case. Fig 4C shows a failure to assemble when only the two negative RNA effects are applied, with no oligomer larger than a dimer ever appearing. Fig 4D shows that the combination of positive effects yields a profile in which elongation-like growth appears almost immediately without a discernible lag phase or nucleation step, leading to the formation of multiple large oligomers. While some manage to combine, the system ends up in a kinetically trapped end state with two large oligomers, a 73mer and a 17mer, that are unable to combine. Additional scenarios are considered in the subsection "Analysis of Twelve Remaining RNA Effect Combinations" below. As with the simulated light scattering profiles, each additional combination of effects appears to group qualitatively with one of the four representative scenarios in Fig 4.

To more clearly illustrate these virtual single-particle assembly pathways, we have also constructed movie files for the four representative scenarios. S1 Movie follows the assembly of a hollow CCMV capsid as in Fig 4A. The movie shows a process of extensive trial-and-error, consistent with the weak bonding mechanisms predicted by numerous prior simulation studies as a way of ensuring nucleation-limited growth [2,7,63,64]. A distinct nucleation step is clearly observed, but in an unexpected way. While we would expect that a stable oligomer would be the key to touching off nucleation, stable pentamer oligomers do form in the simulation but do not make effective templates for further polymerization. Elongation is touched off only when two of these pentagon structures bond together and subsequently close to form a 20-mer containing a hexagon structure, which provides an effective template for continued low-order elongation reactions. Manual examination of other trajectories showed that this specific oligomerization is only one of a diverse array of pathways visualized, however, with nucleation seemingly not dependent on a specific size of intermediate but instead on a specific hexagon substructure, which could first appear as part of a variety of intermediate sizes. Representative frames from this movie are shown in S2 Fig.

S2 Movie follows assembly of a model capsid under combined RNA effects. Relative to S1 Movie, the first striking difference is how much faster assembly is both with respect to simulation time and number of events along the path to assembly completion. The mechanism is both faster and more directed, largely because the far slower off-rate reduces the trial-and-error process seen in the previous movie. Nucleation occurs just following 0.04 seconds as the first hexagon is closed, yielding a 20mer that quickly binds with another 20mer. While binding of large oligomers is still rare, they occur much more frequently in this scenario, as quantified in the next section. Representative frames from this movie are shown in S3 Fig.

S3 Movie shows a trajectory of unsuccessful assembly under the two negative RNA effects, failing to produce any oligomer larger than a trimer on the timescale examined. The high ratio of off- to on-rates means that small oligomers are far more likely to fall apart than grow, making formation of a stable nucleus too rare to observe on this timescale. Representative frames from this movie are shown in S4 Fig. S4 Movie shows combined positive effects, yielding even more rapid initial stages of assembly than the combined RNA effects case. The rapid nucleation, however, leads to two large intermediates that are sterically incompatible but unable to break apart, locking the system into a kinetically trapped state. Representative frames from this movie are shown in S5 Fig.

#### Averaged pathway usage across trajectories

**Fig 5:**
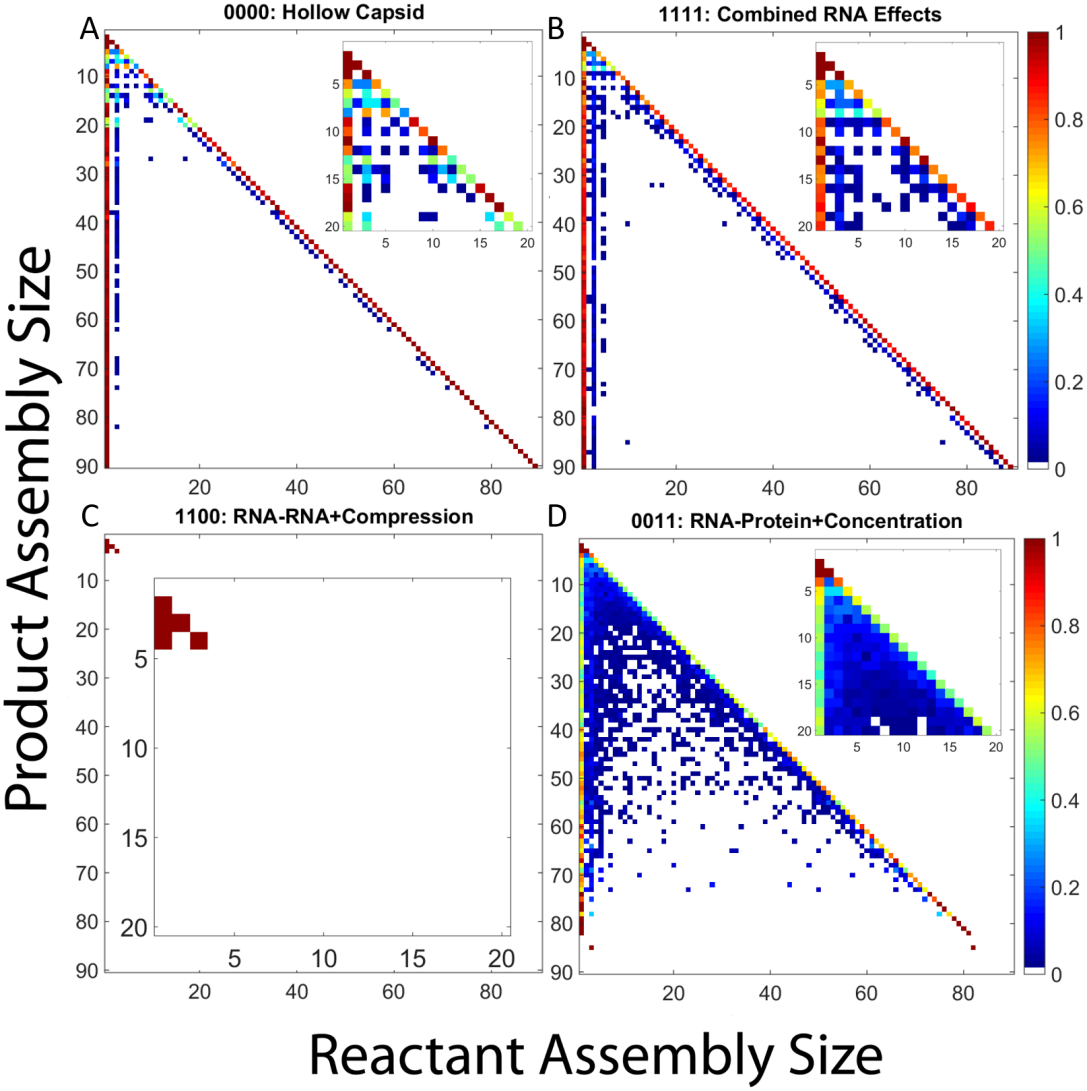
Frequency matrix plots for representative combinations of RNA effects. Frequency matrix plots for (a) hollow CCMV capsid assembly, (b) CCMV capsid assembly with all combined RNA effects, (c) CCMV capsid assembly under both negative RNA effects (1100), and (d) CCMV capsid assembly under both positive RNA effects. In each plot, each row corresponds to a product size and each column to reactant sizes that produce that product. Pixel color in each position corresponds to the frequency with which the given reactant size is used to produce the given product size. Insets within each plot expand the upper-left corner of the main plot, corresponding to products of size 20 or smaller, to better visualize pathways involved in production of small oligomers.

To further understand the effect of RNA on CCMV assembly pathways, we constructed frequency matrix plots quantifying average pathway usage for each potential combination of RNA effects. For each effect combination, the corresponding frequency matrix plot shows the frequency with which each assembly size is used as a reactant in producing any given larger assembly size (e.g., the frequency with which a dimer is a reactant for a reaction producing a pentamer). Fig 5 presents frequency matrix plots for the four effect combinations cases detailed above: hollow capsid (Fig 5A), combined RNA effects (Fig 5B), negative effects only (Fig 5C) and positive effects only (Fig 5D). The remaining cases are considered in subsection "Analysis of Twelve Remaining RNA Effect Combinations" below.

Hollow capsid assembly (Fig 5A) shows two major kinds of elongation reaction: a dominant monomer-addition reaction and a rarer trimer-addition alternative. Assembly under combined RNA effects (Fig 5B) shows both an increase in use of trimer reactions and the emergence of a third pathway of pentamer-addition reactions. Early stages of assembly yield more complex combinations of oligomer reactions for both conditions, but again with a wider variety in the presence of all RNA effects. Fig 5C shows near complete abolition of assembly, with all reactions using only monomer and dimer reactants, in the presence of the negative effects only. Over the two hundred simulations run, the largest intermediate ever formed is a tetramer. The combination of positive effects (Fig 5D) once again yields a far more complex picture involving a diverse array of nearly every potential binding reaction for producing small- to-medium oligomers but total loss of complete or near-complete assemblies. This profile is typical of kinetically trapped domains, where numerous oligomers form and interact but eventually get locked into a domain of sterically incompatible, irreversible partially-assembled forms.

### Analysis of Twelve Remaining RNA Effect Combinations

#### RNA effects on bulk assembly kinetics

**Fig 6:**
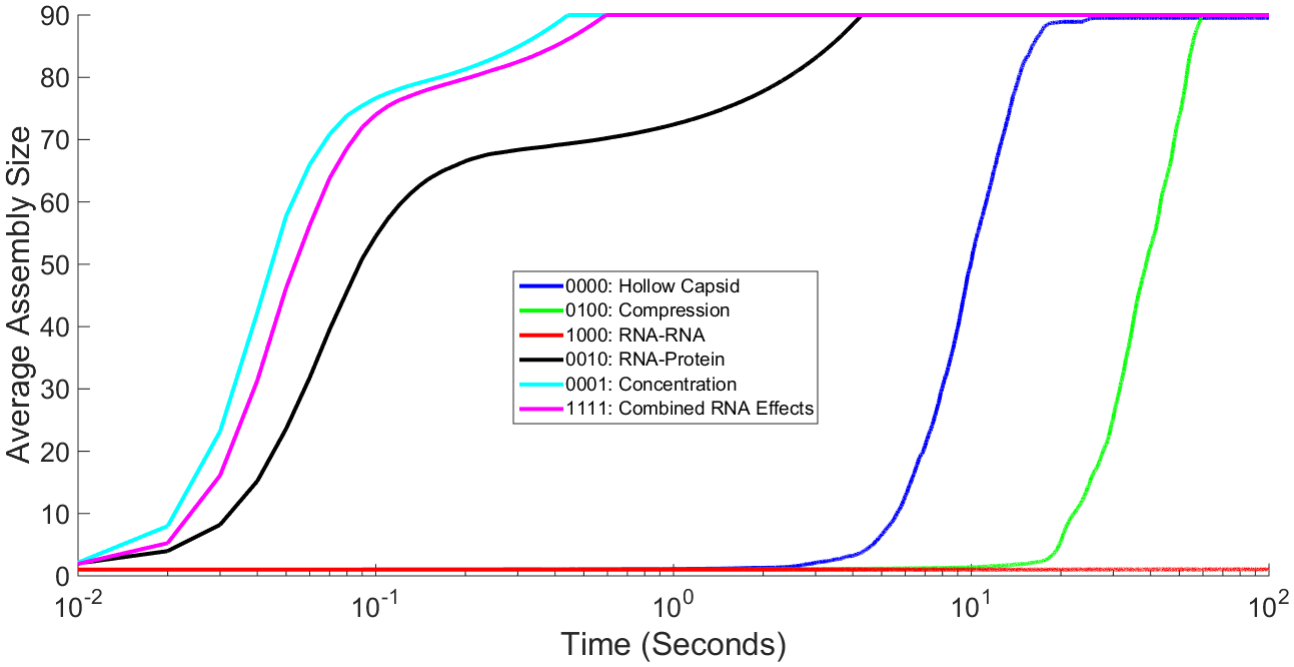
Comparing Influence of Individual RNA Effects on Averaged Assembly Rate. Simulated light scattering curves for CCMV capsid assembly under each individual RNA effect as well as the hollow capsid and combined RNA effects case. Fig 6A shows the entire simulation time course while Fig 6B shows the first second. Time on the x axis is shown on a log scale.

**Fig 7:**
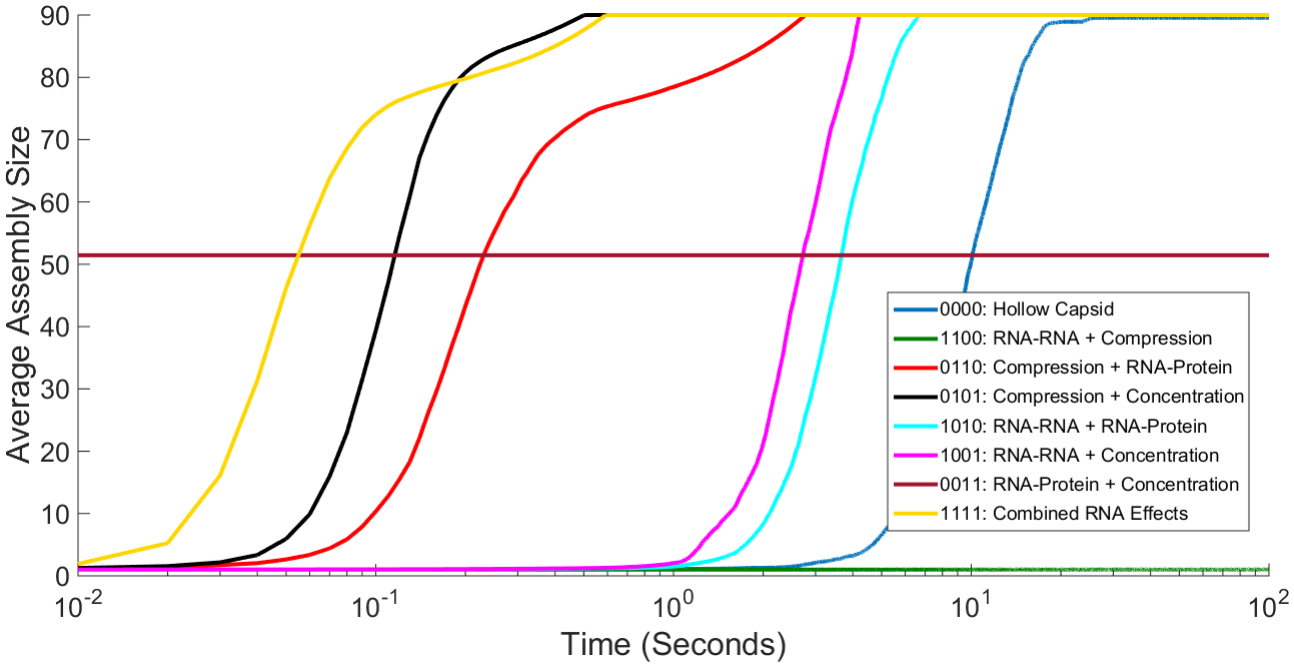
Comparing Influence of Two RNA Effects on Averaged Assembly Rate. Simulated light scattering curves for CCMV capsid assembly under all combinations of two RNA effects as well as the hollow capsid and combined RNA effects case. Fig 7A shows the entire simulation time course while Fig 7B shows the first five seconds. Time on the x axis is shown on a log scale.

**Fig 8:**
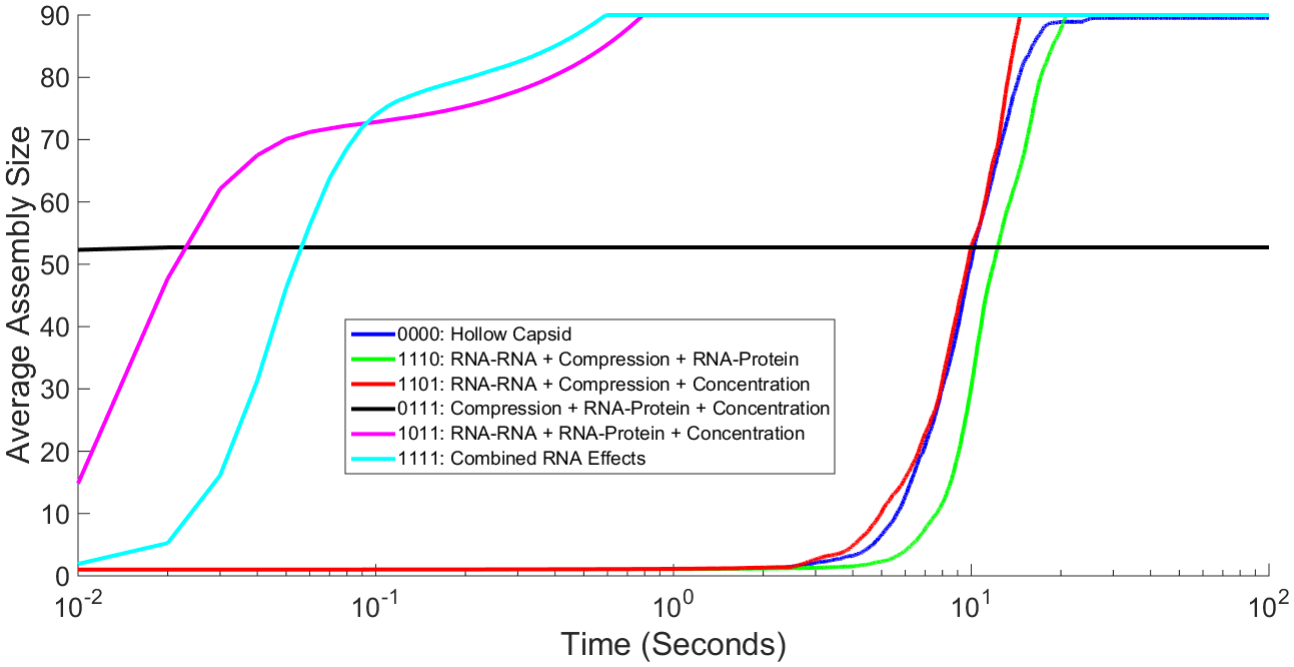
Comparing Influence of Three RNA Effects on Averaged Assembly Rate. Simulated light scattering curves for CCMV capsid assembly under all combinations of three RNA effects as well as the hollow capsid and combined RNA effects case. Fig 8A shows the entire simulation time course while Fig 8B shows the first second. Time on the x axis is shown on a log scale.

Figs 6–8 show simulated light scattering curves for CCMV under different combinations of RNA effects. Each part of the figure includes the cases of no effects as well as all effects to better understand the transition between hollow capsids and the presence of RNA. Because of the large difference in time scales between reactions, each plot is shown in two versions showing a slow timescale (part A) and a fast timescale (part B). Fig 6 examines each effect individually (RNA-RNA, Compression, RNA-protein, and Concentration) as well as curves representing a hollow capsid and a capsid under all RNA effects for comparison. Fig 6A shows the full 100 seconds of simulation and Fig 6B shows just the first 1 second. As expected given the scaling factors of the on- and off-rates (Table 1), Compression moderately reduces the speed of capsid assembly, although assembly is still achieved. RNA-RNA alone prevents any large intermediates from being formed, with nothing above an 8mer assembled in any simulation run. On the other hand, RNA-protein and Concentration both dramatically increase the rate of capsid assembly. Both of these simulated curves show similar kinetics to the combined RNA effects curve, which also shows a far faster assembly rate than the hollow capsid curve.

Fig 7 examines each combination of two effects, as well as our baseline curves for hollow capsid and combined RNA effects, with Fig 7A showing 100 seconds and Fig 7B just the first five seconds. The majority of combinations behave as one would expect just based upon the scaling factors provided in Table 1. Addition of the negative factors of Compression and RNA-RNA slows down assembly rate whereas the addition of the positive factors RNA-protein Concentration increases assembly rate. The combination of Compression and RNA-RNA still fails to produce larger intermediates, with tetramers now being the largest product assembled in our simulation runs. It is interesting to note that although the combination of RNA-RNA and Concentration decreases equilibrium constants for assembly reactions, the overall assembly process is still faster than that of a hollow capsid. This is likely due to the different ways in which these effects are applied. This combination involves speeding up both on- and off-rates and while increasing the off-rate does decrease the stability of assembled products, increasing the on-rate does more than enough to offset that. One surprising combination is that of RNA-protein and Concentration. The two positive effects on capsid assembly, when combined, abolish assembly of capsids in our simulations. While large intermediates can sometimes be constructed, increasing the on-rate to the extent of this combination while also decreasing the off-rate inevitably leads to kinetically trapped intermediates without the ability to reform into completed capsids. This is an example of the complexity and sometimes counter-intuitive nature inherent in self-assembly systems. Combining one positive and one negative effect results in systems that reliably go to completion, with each scenario yielding kinetics between those of the empty capsid and all-effects models. The one partial exception is the combination of Compression with Concentration increase, which shows an initially greater lag than the all-effects model but ultimately goes to completion slightly faster than the model of all four effects.

Fig 8 then examines each combination of three effects as well as the baseline curves. Fig 8A again shows 100 seconds of simulation time, while Fig 8B shows just the first second. In each case where two negative effects are combined with a single positive effect, RNA-RNA + Compression + RNA-protein and RNA-RNA + Compression + Concentration, the simulated curve is very similar to that of the hollow capsid. Once again, combining RNA-protein and Concentration effects without a strong negative effect included still cannot yield completed capsids, shown here in the case of Compression + RNA-protein + Concentration; however, when combined with RNA-RNA, capsids are once again assembled. It should be noted that this combination of three effects, while having higher equilibrium constants for the assembly reactions and a faster initial rate of assembly, requires a longer average time to assemble capsids to completion than does the combined effects case.

#### RNA effects on individual assembly trajectories

**Fig 9:**
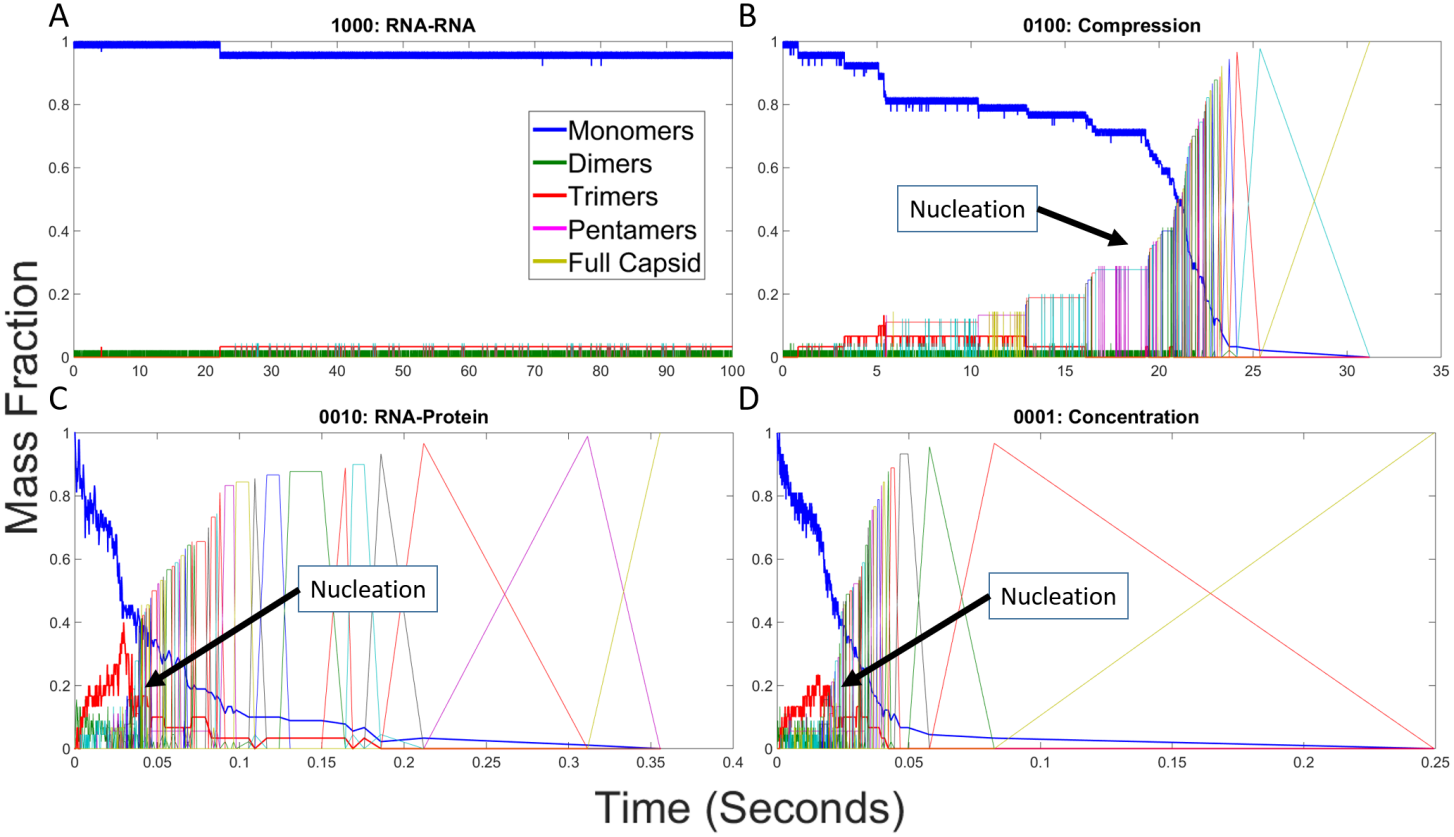
Mass fraction plots for CCMV capsid assembly with individual RNA effects. Mass fraction plots for single trajectories of CCMV capsid assembly upon applying each effect: (A) RNA-RNA, (B) Compression, (C) RNA-protein, (D) Concentration. The negative RNA-RNA effect prevents any large intermediates from being formed, while the other three still allow for capsids to be completed.

**Fig 10:**
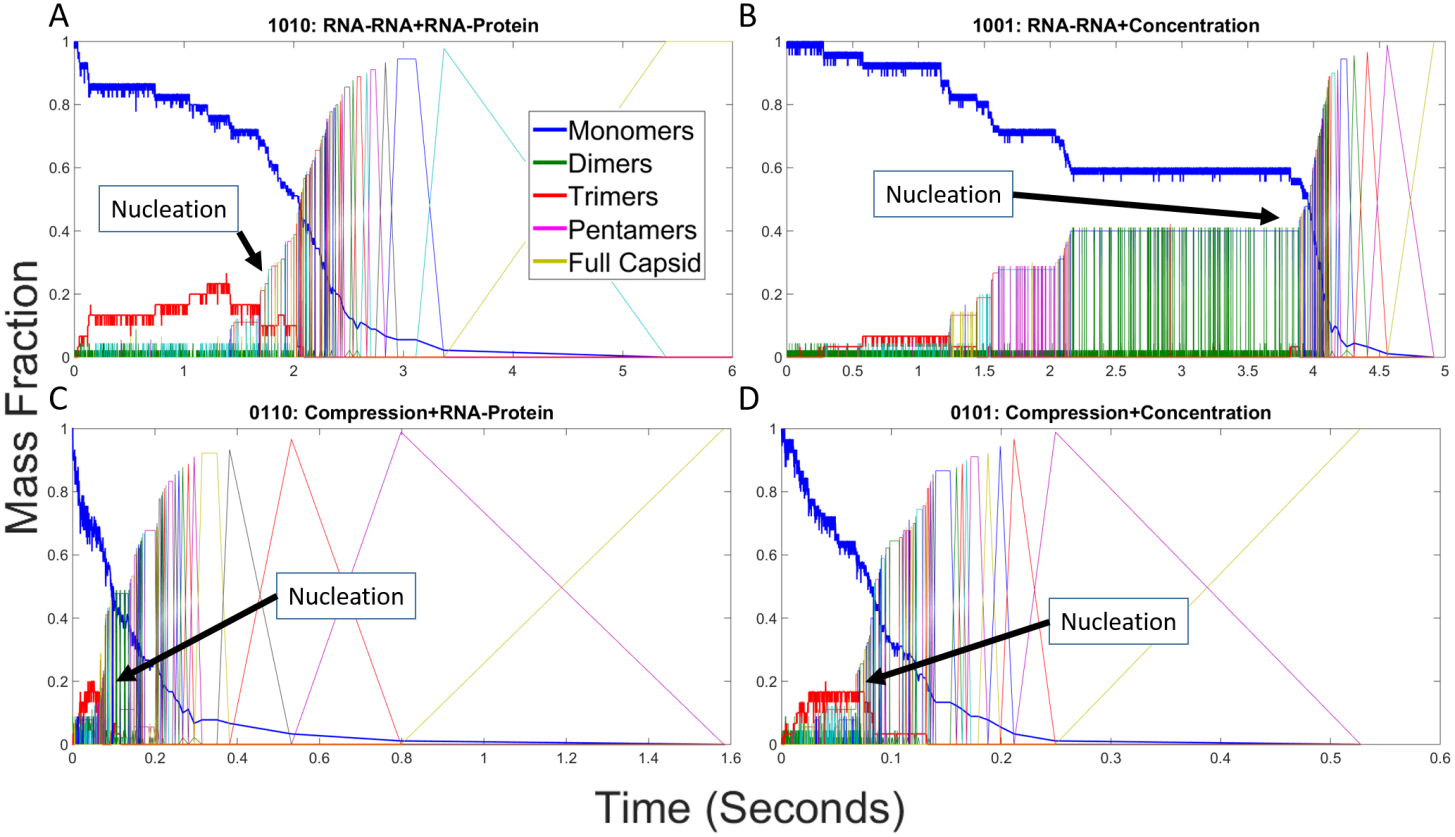
Mass fraction plots for CCMV capsid assembly with combinations of two RNA effects. Combinations are described by a four digit binary code as explained in Table 1, where a 1 means an effect has been turned on and a 0 means an effect has been turned off. The first digit represents RNA-RNA, the second Compression, the third RNA-protein, and the fourth Concentration. (A) is 1010, (B) is 1001, (C) is 0110, (D) is 0101.

**Fig 11:**
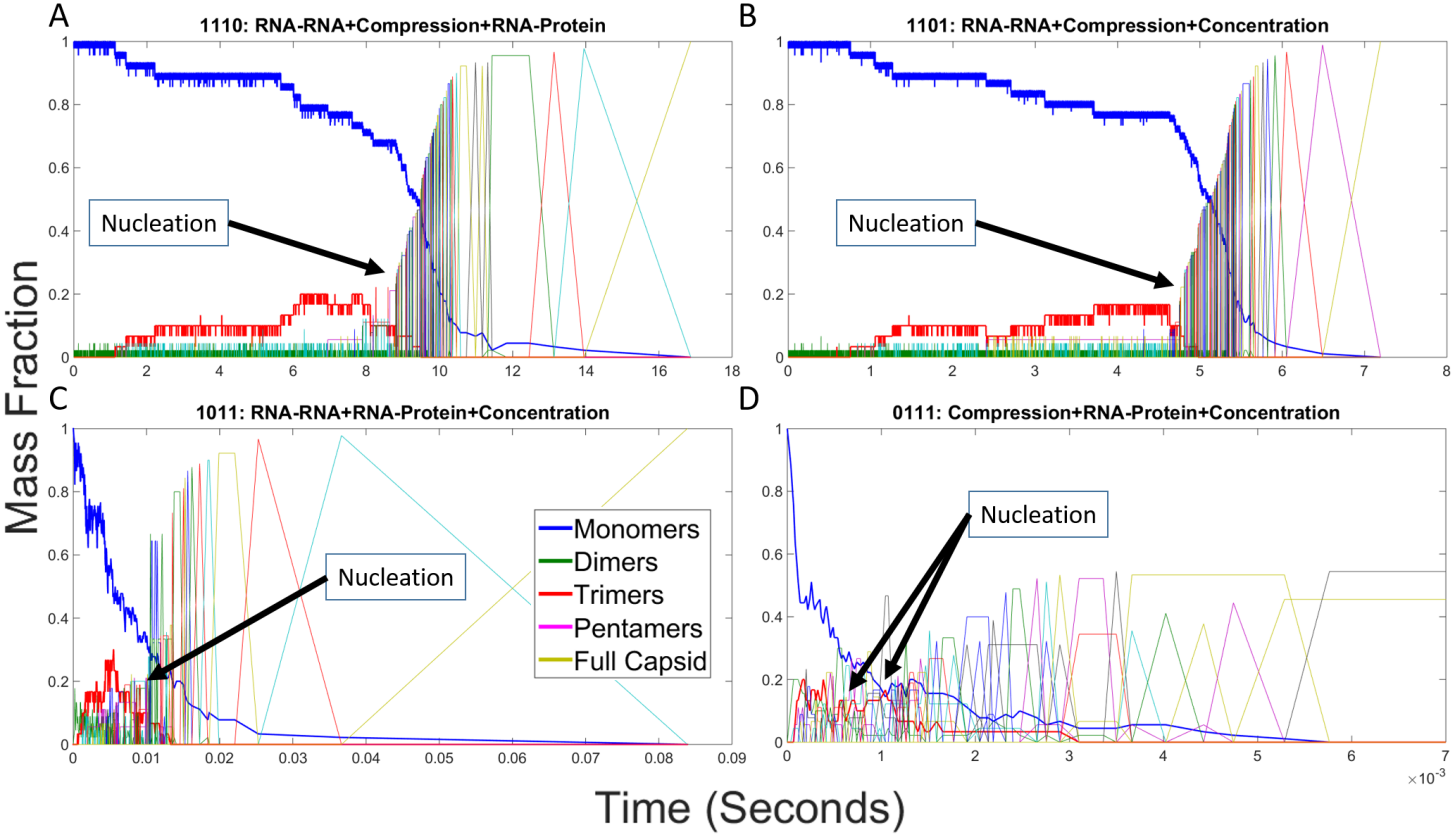
Mass fraction plots for CCMV capsid assembly with combinations of three RNA effects. Combinations are described by a four digit binary code as explained in Table 1 where a 1 means an effect has been turned on and a 0 means an effect has been turned off. The first digit represents RNA-RNA, the second Compression, the third RNA-protein, and the fourth Concentration. (A) is 1110, (B) is 1101, (C) is 1011, (D) is 0111.

We next examine individual simulations to explore fine details of pathway usage in single trajectories. Figs 9–11 provide mass fraction plots showing evolution of counts of distinct oligomer sizes versus time for the twelve scenarios omitted from the previous analysis.

Fig 9 provides mass fraction plots showing trajectories for each individual RNA effect in isolation. Fig 9A shows the RNA-RNA effect, which prevents all larger oligomers from forming. Only one trimer forms during the simulation. Some tetramers transiently form but are unstable and break down before they can seed further growth. In each of the other three cases, capsids still form, though there are some important distinctions. In Fig 9B, the negative effect of Compression slows down overall assembly rate as well as greatly extending the length of the early stages of capsid assembly following the nucleation event. Fig 9C and 9D show a similar assembly process for the positive effects of RNA-Protein and Concentration respectively. In both of these cases, the nucleation rate and overall assembly rate are greatly increased both in absolute terms and relative to the elongation rate.

Fig 10 examines the influence of pairs of RNA effects on assembly, with each subfigure covering one scenario pairing a positive and a negative effect. For each of these figures, we use the same binary four digit code to explain the effect combinations as described in Table 1. The first digit represents RNA-RNA, the second digit represents Compression, the third digit represents RNA-protein and the fourth digit represents Concentration. A value of 1 means that effect is turned on and a value of 0 means that effect has been turned off. Fig 10A shows effect combination 1010, which is RNA-RNA combined with RNA-protein, which behaves similarly to hollow capsid assembly, but with a moderately increased rate of assembly and a slightly more gradual relative assembly rate following the nucleation event. Fig 10B shows effect combination 1001, RNA-RNA combined with Concentration. Again assembly rate is moderately increased over the hollow capsid case, but with significantly altered behavior. Here, again, there is a distinct lag in the earlier stages of assembly following the nucleation event, indicative of a need for a second high-order nucleation-like event to allow elongation to proceed. Oddly, progress seems to depend on interaction of a 36mer with a trimer, a reaction step that we observed frequently, but not always, in simulation runs under these conditions where an apparent second lag occurs. This same point in assembly seems to be important in the similar case of Fig 9B where a lag is seen until assembly of a 39mer. Fig 10C and 10D show similar mass fraction plots to one another. In each case, Compression is paired with a single positive effect, RNA-protein (0110) and Concentration (0101) respectively. Both behave as slightly slowed down versions of the single positive effect plots in Fig 9C and 9D, respectively. This minor change appears consistent with the weaker nature of the Compression effect.

Fig 11 examines the remaining cases, combinations of three different RNA effects, using the same binary code as described previously. Fig 11A and 11B each show both negative RNA effects combined with one of the two positive RNA effects, RNA-protein (1110) or Concentration (1101). In both cases, the mass fraction plot is similar to that of the hollow capsid, although at a slightly faster rate. On the other hand, the effect combination 1011 of RNA-RNA and the two positive effects of RNA-protein and Concentration in Fig 11C, behaves much more like the combined effects case in Fig 3B, although the assembly rate is still faster and the nucleation peak is still much sharper, as with the hollow capsid case. Lastly, Fig 11D shows the effects combination 0111 involving Compression, RNA-protein and Concentration. The figure shows kinetic trapping, similar to what is observed in the case of only positive effects, with elevated on-rates resulting in multiple nucleation events happening nearly simultaneously. Without a fast off-rate to counterbalance these effects, two stable intermediates are formed, a 49mer and a 41mer.

#### Averaged pathway usage across trajectories

We next examine aggregate pathway usage for the twelve scenarios omitted from the preceding analysis, as quantified in frequency matrix plots. Each table shows, for a given scenario, the frequency with which each possible reactant size is used to make each possible product size, averaged over 200 trajectories.

**Fig 12:**
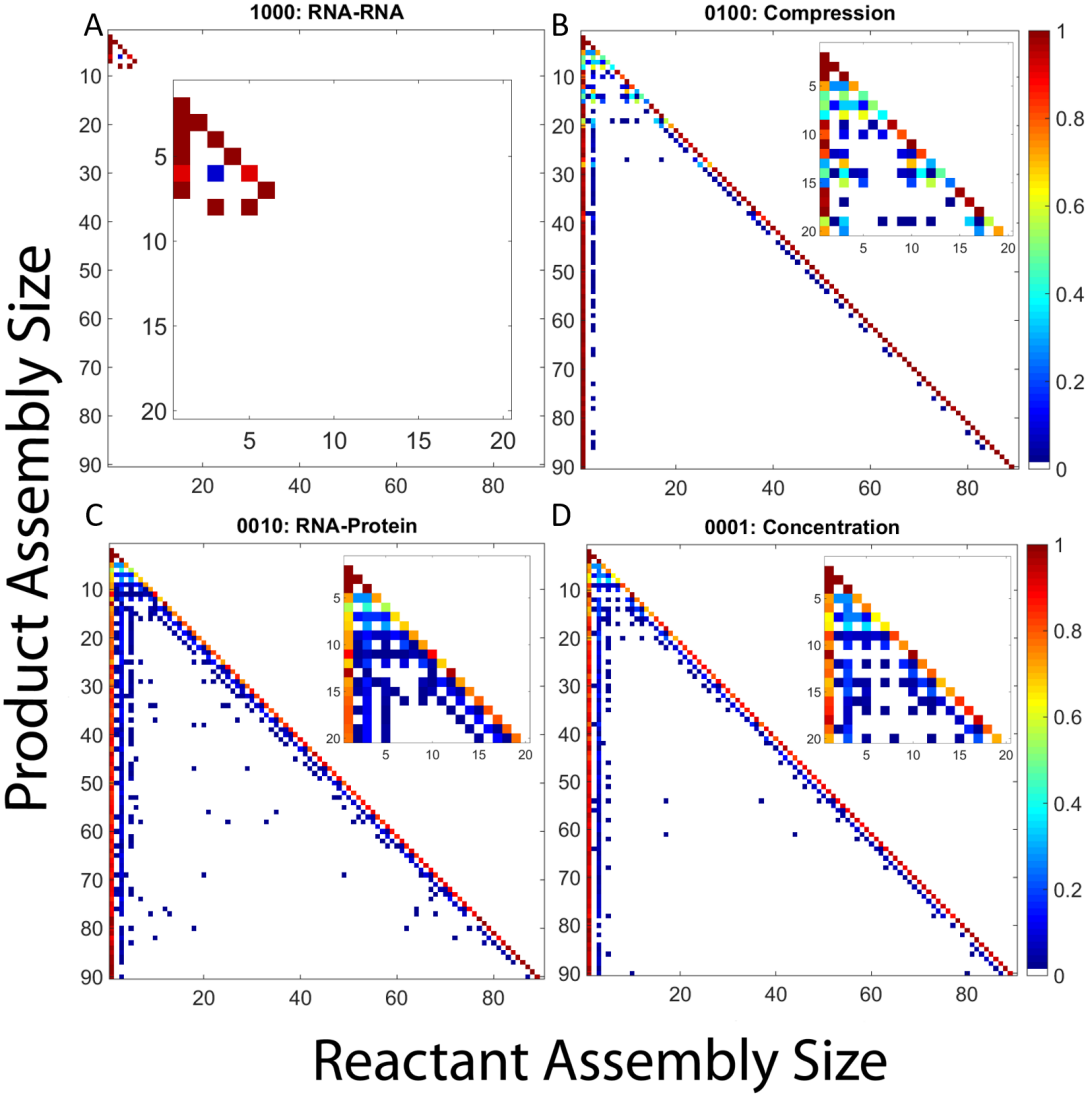
Frequency matrix plots for CCMV capsid assembly with individual RNA effects. Frequency matrix plots averaged over 200 simulation runs for CCMV capsid assembly upon applying: (A) RNA- RNA, (B) Compression, (C) RNA-protein, (D) Concentration. In each plot, each row corresponds to a product size and each column to reactant sizes that produce that product. Pixel color in each position corresponds to the frequency with which the given reactant size is used to produce the given product size. Insets within each plot expand the upper-left corner of the main plot, corresponding to products of size 20 or smaller, to better visualize pathways involved in production of small oligomers.

Fig 12 provides frequency matrix plots for CCMV capsid assembly under single RNA effects. Fig 12A shows once again that RNA-RNA alone abolishes capsid production. Over the two hundred simulation runs for this case, a 8mer was the largest assembly produced. In contrast, the Compression case in Fig 12B exhibits a very similar set of pathways to the hollow capsid case, which is to be expected since Compression has only a minor negative effect on the on rates. Fig 12C and 12D show the two positive effects, RNA-protein and Concentration, both of which behave similarly and more in line with the combined RNA effects of Fig 5B. In both Fig 12C and 12D the pentamer-based assembly pathway is prominent, although the RNA-protein interaction case shows an increased variety in pathways utilized compared to both the Concentration and the combined effects cases.

**Fig 13:**
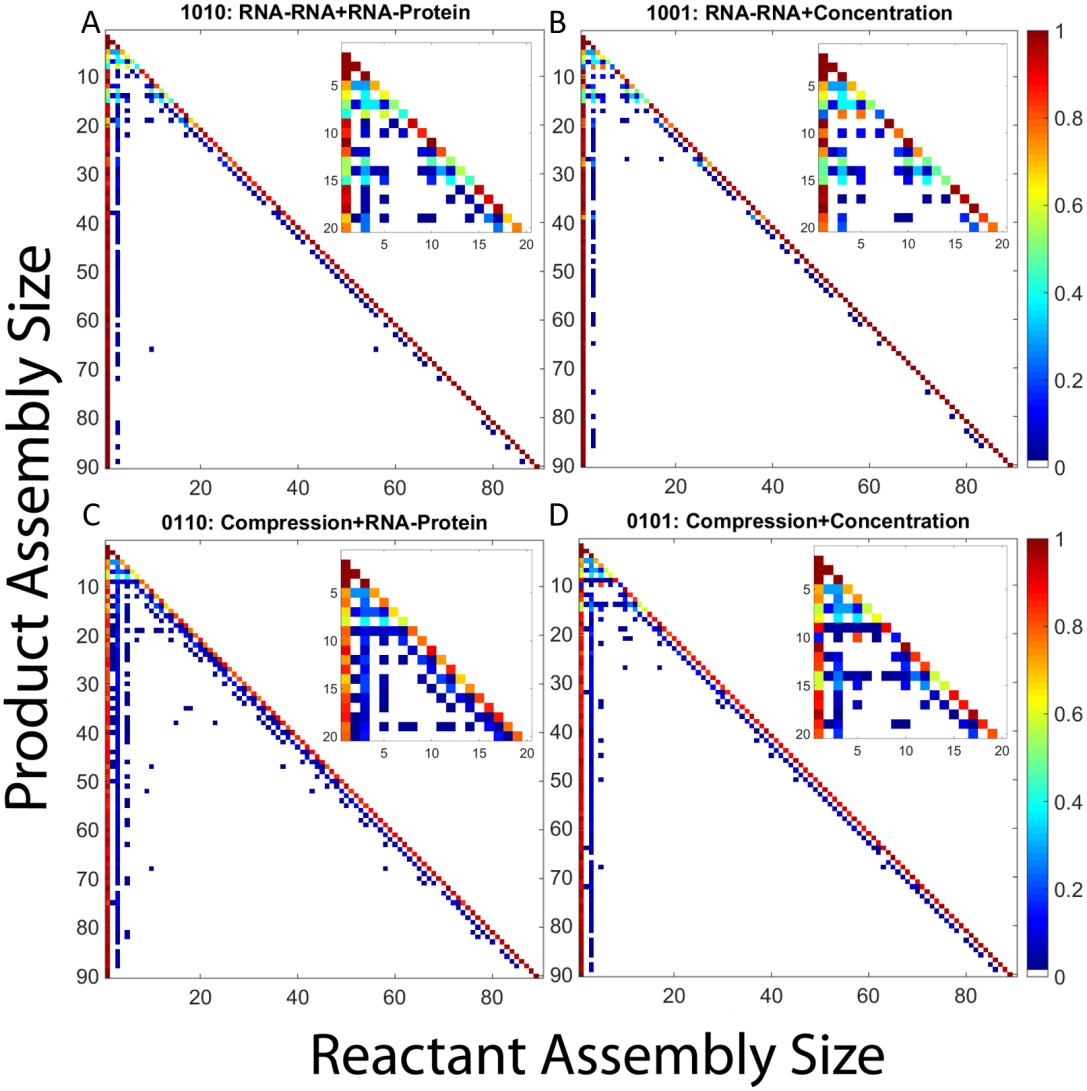
Frequency matrix plots for CCMV capsid assembly with combinations of two RNA effects. Combinations are described by a four digit binary code as in Table 1 where a 1 means an effect has been turned on and a 0 means an effect has been turned off. The first digit represents RNA-RNA, the second Compression, the third RNA-protein, and the fourth Concentration.: (A) is 1010, (B) is 1001, (C) is 0110, (D) is 0101. In each plot, each row corresponds to a product size and each column to reactant sizes that produce that product. Pixel color in each position corresponds to the frequency with which the given reactant size is used to produce the given product size. Insets within each plot expand the upper-left corner of the main plot, corresponding to products of size 20 or smaller, to better visualize pathways involved in production of small oligomers.

Fig 13 shows frequency matrix plots for CCMV capsid assembly under two RNA effects. We again use the same coding scheme as we did for the mass fraction plots and Table 1. Coupling RNA-RNA with either single positive effect produces frequency matrix plots very similar to that of a hollow capsid. Fig 13A and 13B, show the combination of RNA-RNA with RNA-protein (1010) and Concentration (1001), respectively. Both only have two main elongation pathways: monomer and trimer addition. It is interesting to note that there is little difference in the frequency matrix plots despite a significant difference in their mass fraction plots. There is no change in binding frequencies that would seem to provide insight into the second lag phase seen in the 1001 effect combination. Fig 13C, Compression and RNA-protein (0110), and Fig 13D, Compression and Concentration (0101), show similar pathway utilization to Fig 12C and 12D which again makes sense as RNA compression has only a minor negative effect on the on-rate.

**Fig 14:**
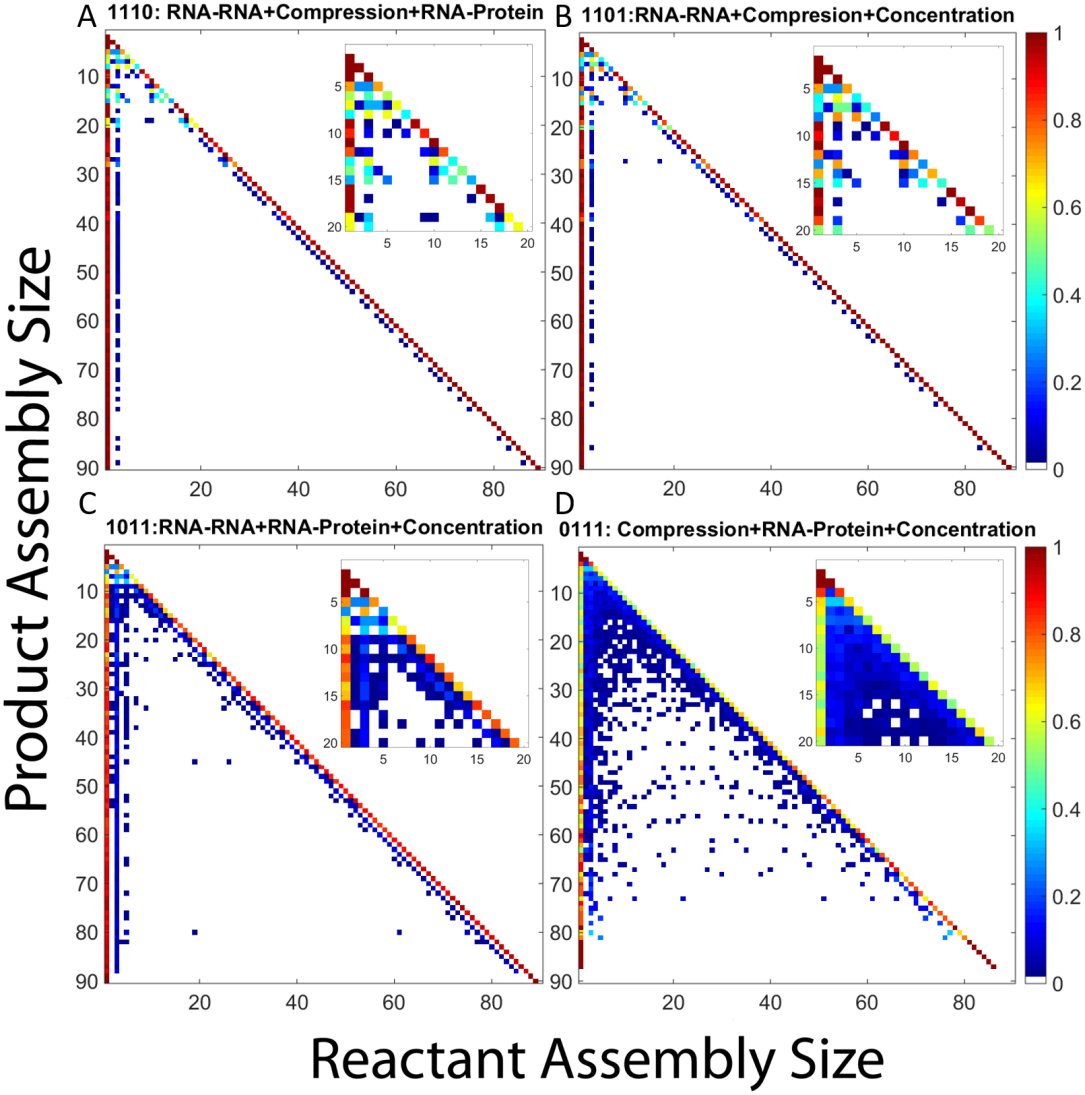
Frequency matrix plots for CCMV capsid assembly with combinations of three RNA effects. Combinations are described by a four digit binary code as in Table 1 where a 1 means an effect has been turned on and a 0 means an effect has been turned off. The first digit represents RNA-RNA, the second Compression, the third RNA-protein, and the fourth Concentration. (A) is 1110, (B) is 1101, (C) is 1011, (D) is 0111. In each plot, each row corresponds to a product size and each column to reactant sizes that produce that product. Pixel color in each position corresponds to the frequency with which the given reactant size is used to produce the given product size. Insets within each plot expand the upper-left corner of the main plot, corresponding to products of size 20 or smaller, to better visualize pathways involved in production of small oligomers.

Fig 14 displays frequency matrix plots of CCMV capsid assembly under combinations of three RNA effects, again with the same binary code previously described. Fig 14A and 14B show combinations involving both negative effects and a single positive effect: RNA-RNA, Compression, and RNA-protei (1110) or RNA-RNA, Compression, and Concentration (1101), respectively. In each case, the frequency matrix plot shows pathways similar to those of the original hollow capsid case with growth primarily based upon monomer and trimer addition. In contrast, Fig 14C shows the combination of RNA-RNA, RNA-protein and Concentration (1011), which yields pathways very similar to the combined effects case, with potentially slightly more diversity in the lower assembly sizes. Lastly, Fig 14D shows the combination of Compression, RNA-protein and Concentration (0111), which is still unable to produce completed capsids but again shows a wide array of binding reactions as was seen in our other kinetically trapped case, the combination of just the two positive effects.

The results of the frequency matrix plots can be grouped into four major potential outcomes. There are two cases in which no large intermediates are produced and thus no real pathway information gained (1000, 1100). Two further cases result in kinetic trapping with a wide array of binding reactions among small-to-medium oligomers but failure to produce complete capsids (0011, 0111). Of the cases where completed capsids are produced, either we see binding reactions which are primarily based on monomer and trimer addition (0000, 0100, 1010, 1001, 1110, 1101) or we see pathways in which a third option of pentamer addition becomes frequent (0010, 1, 0110, 0101, 1011, 1111). The appearance of this third elongation pathway is typically associated with fast, successful assembly while the two-pathway systems tend to assemble more slowly. While this is a simplification, as there is still diversity within each of these classifications, it provides an approximate classification of the range of pathways that can be seen under the different combinations of RNA effects. It is also worth noting that even the simpler pathway sets actually describe ensembles of many possible pathways corresponding to potential addition of multiple possible oligomers and likely multiple possible binding sites at each step of elongation.

## Conclusion

In this work, we have applied a combination of simulation, analytical modeling, and data-fitting to explore how nucleic acid may act to alter kinetics and pathway usage in viral capsid self-assembly, using CCMV assembly on RNA1 as a model system. Using theoretical models of various expected contributions of nucleic acid to assembly, we can make modifications to simulations fit to the RNA-free experimental system to reflect RNA effects without losing the simulation speed or pathway detail essential to these studies. Our approach provides a way to bypass inherent limitations of both purely experimental and purely theoretical studies, using simulation-based model-fitting to learn quantitative models of interactions of purified coat proteins and then computationally project how the activity of those coat proteins might be altered under regimes in which direct experimental observation is infeasible. We break down the effect of the presence of RNA into four categories: RNA-RNA, Compression, RNA-protein, and Concentration increases of coat protein on nucleic acid. The resulting simulations provide an unprecedented level of detail in understanding how nucleic acid might influence fine-scale assembly pathways of a real virus capsid system, revealing potentially significant pathway changes between in vitro and in vivo systems and demonstrating how a balance of growth-promoting and growth-inhibiting forces can act synergistically to greatly accelerate growth without producing the kinetic trapping one would expect from prior theoretical models.

Nucleation-limited growth was long ago established as a key feature of capsid assembly [64] and simulation [7–10] and analytical theory [63,65] have repeatedly suggested that it is crucial to robust capsid production. Nucleation theory provides a conceptual basis for understanding some of our key observations. Our simulations of empty capsids, with parameters learned by direct fitting to in vitro data, clearly show nucleation-limited growth, as we would expect from the relatively weak binding interactions of the learned model and resulting extensive trial-and-error before the capsid can build a stable intermediate. This observation is likewise consistent with prior theory [63–65] and repeated simulation observations [7–10]. It is, then, unsurprising that incorporating those features of nucleic acid that tend to inhibit assembly will abolish growth. The theory predicts that the capsid should exist in a domain in which a rare nucleation event is needed to drive assembly forward, and biasing against growth will make these rare events essentially non-existent on the normal timescale of assembly. It is similarly unsurprising, in the light of nucleation theory, that incorporating only RNA effects that promote assembly likewise abolishes capsid formation. As theoretical studies have long suggested, promoting stronger binding will tend to drive such a system into a kinetically trapped domain, in which excessively rapid nucleation relative to elongation leads to multiple nucleation events before individual capsids can complete, exhausting free subunits and trapping the system in a partially assembled condition, exactly as we observe.

What is interesting, then, is that combining all of the RNA effects leads to growth that is orders of magnitude faster than assembly of the empty capsid, yet just as successful. We would naively expect that a large net shift toward more stable binding would drive the system into the kinetically trapped domain and in fact abolish assembly of completed capsids. Instead, our combined RNA effect model indeed yields much stronger free energies of binding, much faster kinetics, and far less trial-and-error than the empty capsid model, yet it produces capsids with just as much fidelity. This observation corroborates well the findings of Kler et al [35], which learned kinetic rates that fit coarse-grained simulations to x-ray scattering data of capsids assembling around RNA. They found dramatically increased reaction on-rates rapidly pushing assembly to completion in the presence of RNA. In fact, assembly was so rapid, they could find no discernable intermediates present in their experiments. This observation of improved assembly efficiency at such a fast assembly rate is a seeming paradox to prior theoretical models, yet we also know empirically that it is correct. Our results show, for the first time, how the seeming paradox is resolved at the level of fine-scale interactions. Specifically, the RNA effect model produces a striking balance of effects, with positive RNA effects greatly accelerating both pre-nucleation and post-nucleation growth rates while negative effects disproportionately slow prenucleation steps and prevent the multiple-nucleation phenomenon that normally leads to kinetically trapped domains. These trends are reinforced by a novel accumulation of pentamers pre-nucleation that then drive a new pentamer-addition pathway during elongation. Collectively, these effects lead to reliable capsid completion with approximately a 200-fold speedup relative to the empty capsid model.

Our model further suggests the high level of variability the assembly system can tolerate from trajectory to trajectory in the specific pathway to capsid completion. In part, this variability shows up in the stochastic use of different possible oligomer sizes at varying steps during elongation, a variability seen in our earlier empty capsid models [18,23] but enhanced in the present RNA models by the appearance of a pentamer subpopulation contributing to elongation. This stochastic pathway selection also appears in the apparent variability in nucleus structures the model can tolerate, with assembly appearing to require not a specific size of nucleus per se, but rather any combination of oligomers that can combine to form a specific rare, stable substructure.

Like all theoretical work, this study does depend on numerous assumptions and approximations in the simulation models, the process of fitting model parameters to experimental data, and in the analytical theories used to estimate corrections to those parameters to account for the presence of nucleic acid. The computational difficulty of simulating a molecular reaction system on the timescale of capsid assembly makes dramatic simplifications of some form a practical necessity, although different model types may simplify in different ways. The primary assumptions of the stochastic simulation approach used here are 1) that assembly proceeds by individual reactions (associations and dissociations) with all species well-mixed between these reactions and 2) that rates of reactions are determined solely by the specific binding sites involved in those reactions, except where a reaction is precluded by steric hindrance. The fine details of the resulting simulations need to be considered speculative and considered a source of hypotheses rather than a definitive statement about how CCMV assembles on RNA. Nonetheless, it provides projections at a level of detail unavailable by any prior method, revealing emergent effects of the models that would not have been predictable by any other method and revealing a possible answer for an important puzzle in reconciling theory and empirical observation about virus assembly in vitro versus in vivo. While our models do not provide certainty about true CCMV assembly pathways, neither can any other method, and our models provide a better resolution of the potential changes in fine-scale assembly pathways in the two domains than any alternative known to us. We hope these models can inspire future work, beyond the scope of our own theoretical study, to evaluate testable features of these models on real capsid assembly systems and explore potential changes in or relaxations of the specific model assumptions employed here.

In addition to providing insight into an important but puzzling feature of the assembly of RNA viruses, the work helps illustrate some of the continuing value of simulation methods and the synthesis of simulation, experiment, and analytical theory. The simulation approach provides a unique window into fine-scale reaction processes, such as capsid assembly, by providing a platform in which we can observe and precisely quantify aspects of assembly that we have no technology to observe experimentally. Model-fitting to experimental data makes it possible to bring this capability out of the realm of abstract theory of generic capsids to prediction about experimentally unobservable features of specific real systems. Analytical theory lets us take that contribution a step further, to project how the system might behave in environments in which we cannot monitor assembly dynamics experimentally at all. While we have applied those capabilities here to a specific question of how nucleic acid might influence assembly of spherical capsids, this combination of techniques can be expected to apply to numerous other complex systems for which theory, simulation, and experimental methods individually are limited.

## Supporting Information

S1 Fig Box plots of simulation variability in maximum assembly size reached (A) and time to reach respective maximal assembly sizes (B) in log scale for each RNA effect combination. Box plots were generated with the standard inputs for the Matlab boxplot command. Column labels correspond to a binary code for presence (1) or absence (0) of the four effects as in Table 1. The first digit represents RNA-RNA, the second Compression, the third RNA-protein, and the fourth Concentration.
(TIF)

S2 Fig Four representative snapshots from S1 Movie. The first shows the first formation of a trimer. The second shows the formation of a stable 10mer intermediate. The third shows a possible nucleation step at the union of two 10mers. The fourth shows a completed structure.
(TIF)

S3 Fig Four representative frames from S2 Movie. The first shows the first formation of a trimer. The second shows the stable intermediate 10mer with a growing chain of attached trimers. The third shows the formation of a 40mer from two separate 20mer intermediates, an unusually large assembly reaction for these simulations. The fourth shows a completed structure produced on a much faster time scale compared to the hollow capsid assembly.
(TIF)

S4 Fig Two representative frames from S3 Movie. The first shows the first formation of a dimer. The second shows the largest assembly formed in the simulation, a trimer, which took 57 seconds to create.
(TIF)

S5 Fig Four representative frames from S4 Movie. The first shows the first formation of a dimer. The second shows the formation of the stable 10mer structure with a fast-growing series of extensions. The third shows a 39mer that has grown rapidly without much order. The fourth shows the largest assembly created by the simulation, a 73mer, with clear holes in the capsid where rapid bond-forming has resulted in a kinetically trapped intermediate unable to form a completed structure.
(TIF)

S1 Movie Animation of a single trajectory of assembly of a hollow model CCMV capsid in the absence of RNA effects, as in Fig 4A. (MP4)

S2 Movie Animation of a single trajectory of assembly of a model CCMV capsid in the presence of all four RNA effects, as in Fig 4B. (MP4)

S3 Movie Animation of a single trajectory of assembly of a model CCMV capsid in the presence of the two negative RNA effects, RNA-RNA and Compression, as in Fig 4C. (MP4)

S4 Movie Animation of a single trajectory of assembly of a model CCMV capsid in the presence of the two positive RNA effects, RNA-protein and Concentration, as in Fig 4D. (MP4)
