# Supplementary figures and images for: Modeling Effects of RNA on Capsid Assembly Pathways via Coarse-Grained Stochastic Simulation
Source: PLoS One. 2016 May 31;11(5):e0156547. doi: 10.1371/journal.pone.0156547 (PMC4887116; doi:10.1371/journal.pone.0156547)

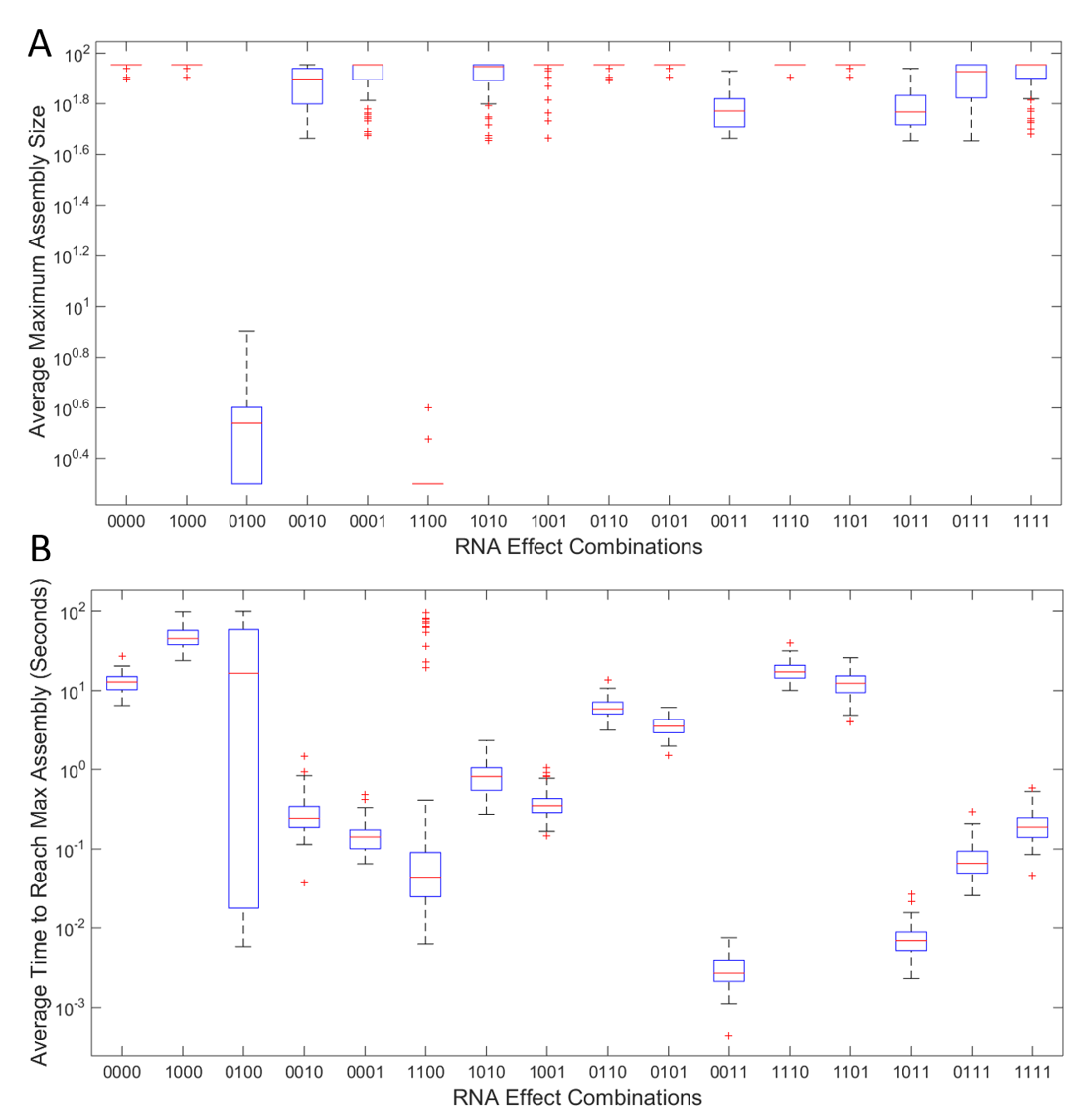

Supplement: S1 Fig — Box plots were generated with the standard inputs for the Matlab boxplot command. Column labels correspond to a binary code for presence (1) or absence (0) of the four effects as in Table 1. The first digit represents RNA-RNA, the second Compression, the third RNA-protein, and the fourth Concentration. (TIF) [file pone.0156547.s001.tif]

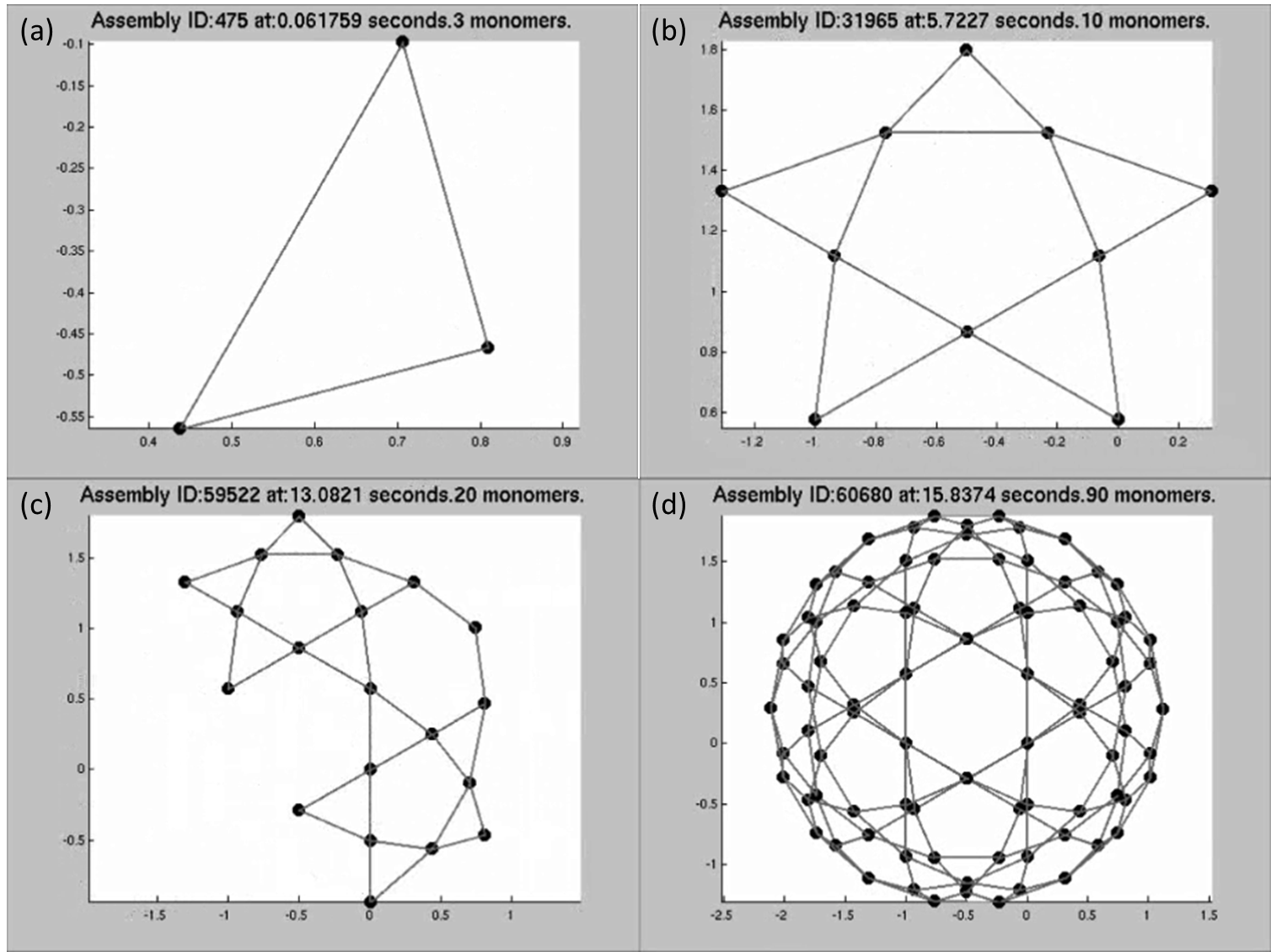

Supplement: S2 Fig — The first shows the first formation of a trimer. The second shows the formation of a stable 10mer intermediate. The third shows a possible nucleation step at the union of two 10mers. The fourth shows a completed structure. (TIF) [file pone.0156547.s002.tif]

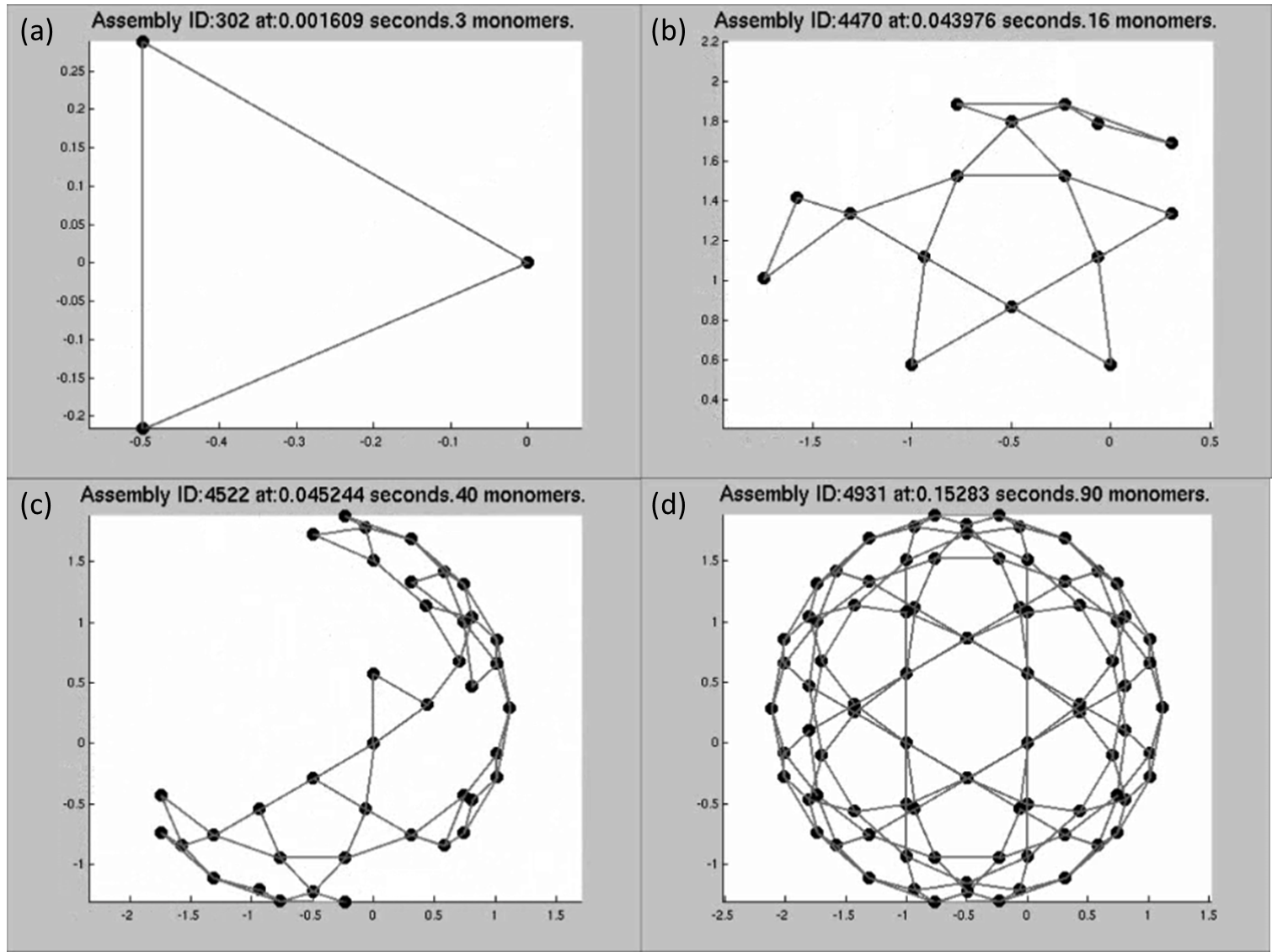

Supplement: S3 Fig — The first shows the first formation of a trimer. The second shows the stable intermediate 10mer with a growing chain of attached trimers. The third shows the formation of a 40mer from two separate 20mer intermediates, an unusually large assembly reaction for these simulations. The fourth shows a completed structure produced on a much faster time scale compared to the hollow capsid assembly. (TIF) [file pone.0156547.s003.tif]

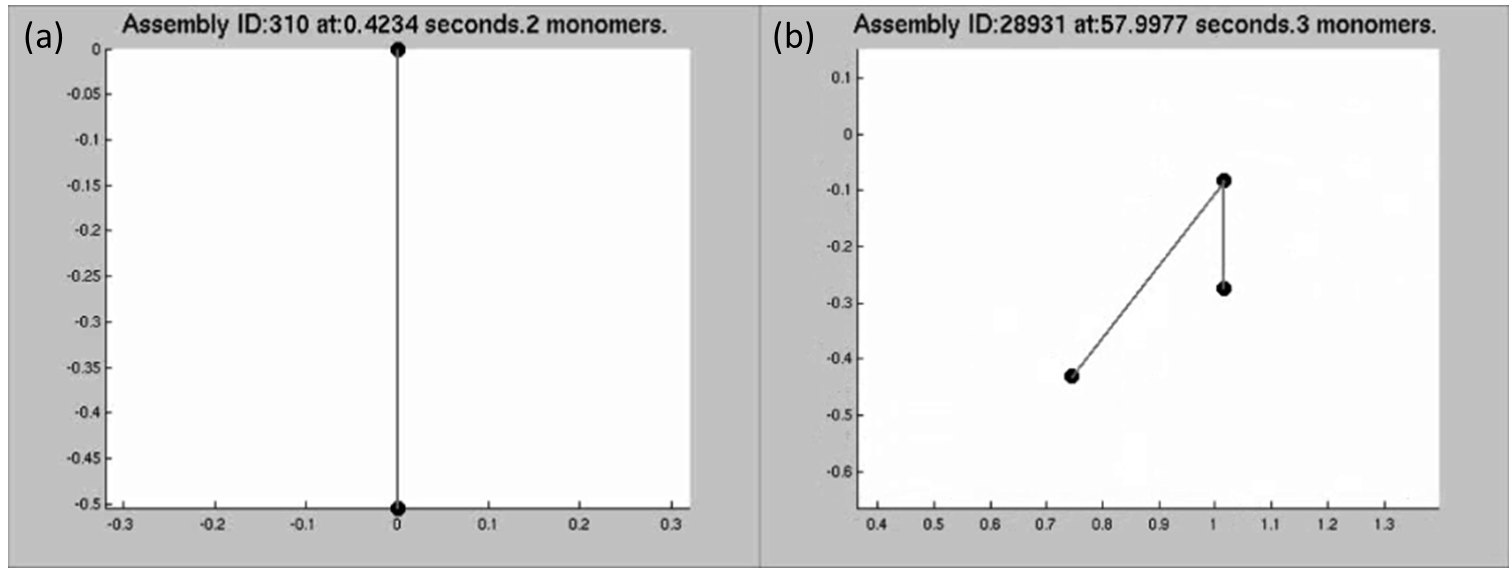

Supplement: S4 Fig — The first shows the first formation of a dimer. The second shows the largest assembly formed in the simulation, a trimer, which took 57 seconds to create. (TIF) [file pone.0156547.s004.tif]

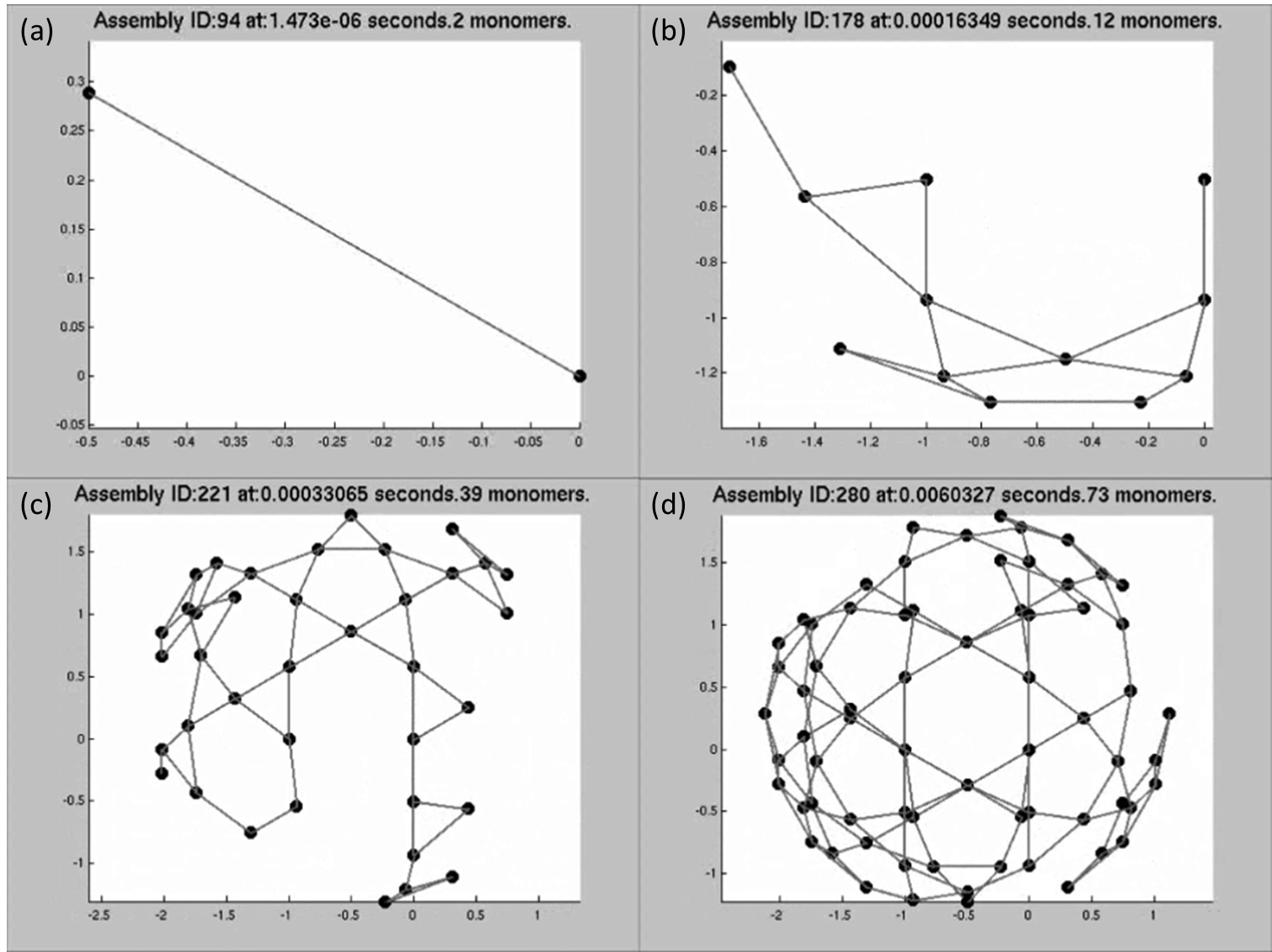

Supplement: S5 Fig — The first shows the first formation of a dimer. The second shows the formation of the stable 10mer structure with a fast-growing series of extensions. The third shows a 39mer that has grown rapidly without much order. The fourth shows the largest assembly created by the simulation, a 73mer, with clear holes in the capsid where rapid bond-forming has resulted in a kinetically trapped intermediate unable to form a completed structure. (TIF) [file pone.0156547.s005.tif]
